# Rapid expansion of primary human vocal fold epithelial cells via targeted pathway inhibition and anchorage-independent sphere culture

**DOI:** 10.1016/j.crmeth.2026.101310

**Published:** 2026-03-06

**Authors:** Xudong Shi, Ryo Suzuki, Haiyan Lu, Hua Zhang, Lingjun Li, Nathan V. Welham

**Affiliations:** 1 Department of Otolaryngology - Head and Neck Surgery, University of Wisconsin-Madison, Madison, WI 53792, USA; 2 Division of Pharmaceutical Sciences, School of Pharmacy, University of Wisconsin-Madison, Madison, WI 53705, USA; 3 Department of Chemistry, University of Wisconsin-Madison, Madison, WI 53706, USA

**Keywords:** *in vitro* techniques, respiratory mucosa, larynx, cellular spheroids, TGF-β inhibition, ROCK inhibition, Notch inhibition, A-83-01, Y-27632, DAPT

## Abstract

Vocal fold epithelial cells (VFEs) serve critical physiologic and immunologic functions at the boundary between the upper and lower airways but are difficult to maintain and expand in primary cultures. This technical challenge has impeded progress in VFE biology as well as cell banking for translational applications. Here, using primary human VFEs, we show that simultaneous inhibition of transforming growth factor β (TGF-β), Rho-associated protein kinase (ROCK), and Notch signaling with a small-molecule inhibitor cocktail enables rapid proliferation, successful passaging, and long-term expansion while preserving the core epithelial phenotype. Under anchorage-independent culture conditions, VFE progenitors generate clonal spheres that can be expanded over multiple generations; sphere-dissociated VFEs then revert toward their original phenotype, which includes the ability to form stratified squamous epithelium in organotypic cocultures. Both pathway-inhibited and sphere-cultured VFEs exhibit mechanistically appropriate remodeling of the cellular proteome. These advances offer a robust toolkit for upper airway mucosal biology and regenerative medicine.

## Highlights

• TGF-β, ROCK, and Notch inhibition drive vocal fold epithelial cell proliferation
• Anchorage-independent culture promotes formation of clonal epithelial spheres
• Pathway inhibition and sphere culture differentially alter the cellular proteome

## Motivation

Primary vocal fold epithelial cells are challenging to culture and expand *in vitro*, hampering progress in vocal fold biology and its translational applications. To address the technical challenges of limited proliferation and growth arrest, we identified and tested a small-molecule inhibitor cocktail targeting TGF-β, ROCK, and Notch signaling pathways and introduced an anchorage-independent culture technique to facilitate formation of progenitor-enriched clonal spheres.

## Graphical abstract

**Figure:**
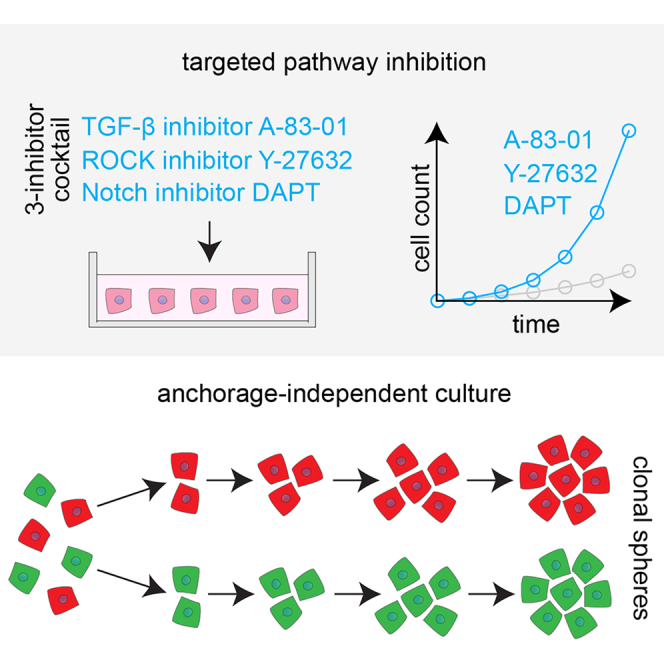

## Teaser

Shi et al. report improved primary culture of human vocal fold epithelial cells by using a small-molecule inhibitor cocktail that targets TGF-β, ROCK, and Notch signaling and by employing a free-floating sphere-formation technique. These complementary methods enable rapid population expansion and progenitor enrichment for research and translational applications.

## Introduction

Housed in the larynx, the paired vocal fold (VF) mucosae underpin vocalization and airway protection in humans and most other terrestrial mammals. The luminal surfaces of these mucosae are lined with VF epithelial cells (VFEs) in stratified squamous formation.^1^ VFEs are essential to mucosal barrier function,^2^^,^^3^^,^^4^ water and ion transport,^5^^,^^6^ upper airway immunology,^7^ and maintenance of microbial eubiosis.^8^ Despite their physiologic importance, however, there are few data characterizing fundamental VFE biology itself. This knowledge gap is perpetuated by a dearth of *in vitro* studies using primary VFEs, which, in turn, is largely due to the technical challenge of isolating, purifying, and expanding these cells in culture.^9^^,^^10^ Such experimental work—especially with disease-free human cells—has been reported by a limited number of specialized laboratories.^11^^,^^12^^,^^13^^,^^14^ The development of more robust and replicable VFE culture methods would advance progress in VF mucosal biology, as well as the translation of emerging therapies that depend on the expansion and banking of clinical-grade cells.

One approach to enhancing primary epithelial cell culture is to modify the cells’ biochemical environment.^15^ Recent evidence has shown that small-molecule inhibitors that target developmental signaling pathways—such as those regulated by transforming growth factor β (TGF-β), Rho-associated protein kinase (ROCK), bone morphogenetic protein (BMP), cyclic adenosine monophosphate (cAMP), Notch, and Wnt—can improve the growth kinetics and ease of passage of epithelial cells isolated from multiple tissues.^16^^,^^17^^,^^18^^,^^19^^,^^20^^,^^21^ Collectively, these inhibitors promote epithelial cell proliferation, suppress apoptosis, and inhibit epithelial-to-mesenchymal transition during prolonged culture. Notably, in work focused on cells isolated from the airway, short-term culture with Notch and ROCK inhibitors enabled primary tracheal epithelial cell expansion while maintaining basal cell differentiation capacity,^18^ whereas prolonged culture with TGF-β and ROCK inhibitors resulted in a 10^12^-fold expansion of primary bronchial epithelial cells over 10 passages with preservation of genomic integrity.^21^

Epithelial cell culture may also be facilitated by physical cues, particularly those that recapitulate elements of the cells’ *in vivo* environment. An example is anchorage-independent culture, in which proliferating epithelial (and other) cells self-organize into three-dimensional (3D) spheres.^22^^,^^23^^,^^24^^,^^25^ These progenitor-enriched spheres can be serially passaged for multiple generations, enabling efficient population expansion and yielding large quantities of progeny cells. Prior reports have shown that epithelial cells can transition from monoculture to sphere culture and that mature spheres can be dissociated into single cells and returned to monoculture.^26^^,^^27^ Sphere culture methods have also evolved, contributing to the development of next-generation organoid platforms that even more closely mimic the *in vivo* environment, enabling detailed investigation of cell-cell and cell-matrix interactions that are otherwise not represented *in vitro*.^28^^,^^29^^,^^30^^,^^31^

Given these promising reports in related systems, we hypothesized that targeted pathway inhibition and anchorage-independent culture techniques would help mitigate the technical challenges that have long curtailed *in vitro* work with VFEs. Using primary human cells, we identified a small-molecule inhibitor cocktail that enabled rapid VFE proliferation, successful culture passage, and long-term population expansion while maintaining the cells’ core epithelial phenotype. We further showed that VFE progenitors can form clonal spheres over multiple generations, that VFE spheres can be returned to monoculture, and that sphere-dissociated VFEs revert toward their original phenotype, including the ability to form stratified squamous epithelium when placed in organotypic co-culture with vocal fold fibroblasts (VFFs). These methodological advances offer a robust toolkit for replicable progress in VFE biology, advanced modeling of VF mucosa, and applications in regenerative medicine.

## Results

### Simultaneous TGF-β, ROCK, and Notch pathway inhibition promotes VFE proliferation while maintaining core epithelial phenotype

**Figure 1:**
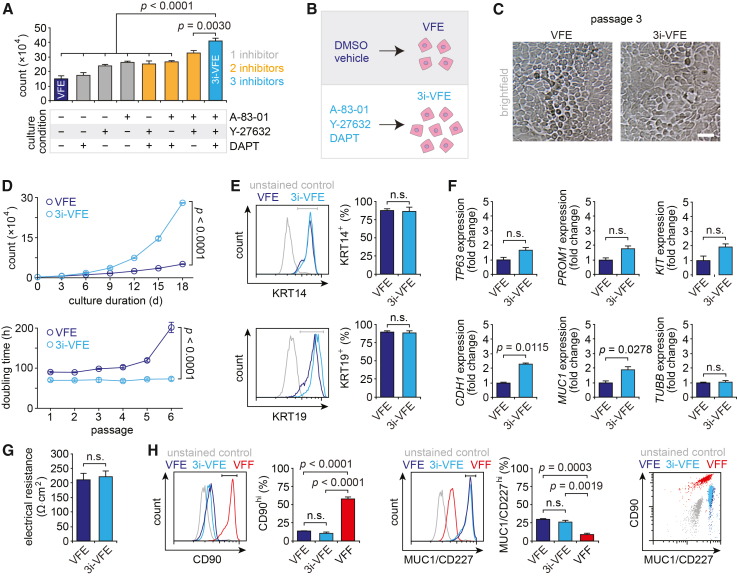
Simultaneous TGF-β, ROCK, and Notch pathway inhibition promotes VFE proliferation while maintaining core epithelial phenotype (A) Effect of candidate small-molecule inhibitors on VFE proliferation. Cells (5 × 10^4^) were incubated with 1 μM A-83-01 (TGF-β inhibitor), 10 μM Y-27632 (ROCK inhibitor), and 5 μM DAPT (Notch inhibitor), as indicated. Counts were performed at day 9; data are plotted as mean ± SEM (*n* = 6); *p* values were obtained using mixed-model ANOVA with planned pairwise comparisons shown. Additional dose-response data for DAPT are presented in Figure S1. (B) Experimental conditions used for subsequent experiments. Cells in the 3i-VFE condition were incubated with a three-molecule cocktail of 1 μM A-83-01, 10 μM Y-27632, and 5 μM DAPT; cells in the VFE condition were incubated with DMSO vehicle. (C) Bright-field images of live cells at passage 3; all cells exhibited cuboidal morphology over serial passages. Scale bar, 20 μm. (D) Single-passage growth curves and population doubling times. Data are plotted as mean ± SEM (*n* = 4); *p* values were obtained using mixed-model ANOVA. (E) Flow cytometry data showing KRT14 and KRT19 expression. Positive/negative gates (versus unstained controls) are shown in gray. Data are plotted as mean ± SEM (*n* = 3); *p* values were obtained using a paired *t* test. (F) RT-qPCR data showing *TP63*, *PROM1*, *KIT*, *CDH1*, and *MUC1* transcription; *TUBB* is reported as an additional reference standard. Data are presented as fold change relative to VFE (mean ± SEM; *n* = 3); *p* values were obtained using a paired *t* test. (G) Transepithelial electrical resistance. Data are plotted as mean ± SEM (*n* = 6); the *p* value was obtained using a paired *t* test. (H) Flow cytometry data showing CD90 and MUC1 (also known as CD227) expression; VFFs are included as a non-epithelial cell control. High/low gates are shown in black. Data are plotted as mean ± SEM (*n* = 3); *p* values were obtained using paired and unpaired *t* tests. Note that the 3i-VFE data (light blue) are largely superimposed on the VFE data (dark blue) in the dot plot.

We used a previously reported approach for primary VFE isolation and culture.^13^ Briefly, human VF mucosae were procured from cadavers at <35 h postmortem; cells were released via enzymatic digestion, and VFEs were isolated and purified using a stepwise adhesion-based protocol. Next, to improve VFE viability and expansion capacity *in vitro*, we screened a panel of small-molecule inhibitors with reported effectiveness in maintaining non-VF primary epithelial cells during extended culture. Pilot dose-response experiments using the TGF-β inhibitor A-83-01, the ROCK inhibitor Y-27632, and the Notch inhibitor DAPT suggested that combination pathway inhibition with all three molecules (hereafter referred to as 3i) supported long-term VFE growth without negative side effects (Figures 1A and S1). We, therefore, assayed growth kinetics and phenotypic stability in primary human VFEs incubated with this three-component inhibitor cocktail (3i-VFE condition) compared to vehicle (VFE condition) (Figure 1B).

Cells in the 3i-VFE condition retained a cuboidal morphology (Figure 1C) and proliferated more rapidly than control cells, resulting in lower population doubling times across serial passages (Figure 1D). 3i-VFE cell doubling times remained stable over 6 passages, whereas VFE doubling times steadily increased, consistent with the onset of cellular senescence in the control condition. Flow cytometry analysis of the phenotypic markers keratin 14 and 19 (KRT14 and KRT19, respectively), previously identified as being expressed by human VFEs *in vivo*^32^^,^^33^ and *in vitro*,^13^ showed no difference across conditions (Figure 1E). Follow-up quantitative reverse-transcription PCR (RT-qPCR) revealed increased transcription of epithelial function-related genes encoding cadherin 1 (*CDH1*; also known as E-cadherin or CD324) and mucin 1 (*MUC1*; also known as epithelial membrane antigen or CD227) in 3i-VFEs compared with VFEs; transcription of genes encoding the progenitor cell markers tumor protein p63 (*TP63*), prominin 1 (*PROM1*; also known as CD133), and KIT proto-oncogene receptor tyrosine kinase (*KIT*; also known as CD117) showed no significant differences (Figure 1F). Transepithelial electrical resistance, a measure of physiologic barrier function, was comparable in 3i-VFEs and VFEs (Figure 1G).

Together, these data indicate that TGF-β, ROCK, and Notch pathway inhibition promotes VFE proliferation, leading to consistent growth kinetics and robust population expansion across at least 6 culture passages. Sustained culture through passage 15 revealed no change in cell behavior or morphology; 3i-VFEs were also successfully cryopreserved and recovered at each passage. To validate our observation that the inhibitor cocktail does not alter the cells’ core epithelial phenotype, we conducted additional flow cytometry to assess phenotypic separation of 3i-VFE and VFE from concurrently isolated VFFs. Applying an established two-marker panel consisting of MUC1/CD227 and the fibroblast marker CD90 (also known as Thy-1),^13^ we separated the cells into CD227^lo^CD90^hi^ (fibroblast) and CD227^hi^CD90^lo^ (epithelial cell) subpopulations, and observed no difference in cell surface marker abundance between the 3i-VFE and VFE conditions (Figure 1H). Of note, the differential *MUC1* expression observed in our RT-qPCR assay (Figure 1F) did not translate to the protein level.

### VFE progenitors generate clonal spheres that can be serially passaged

**Figure 2:**
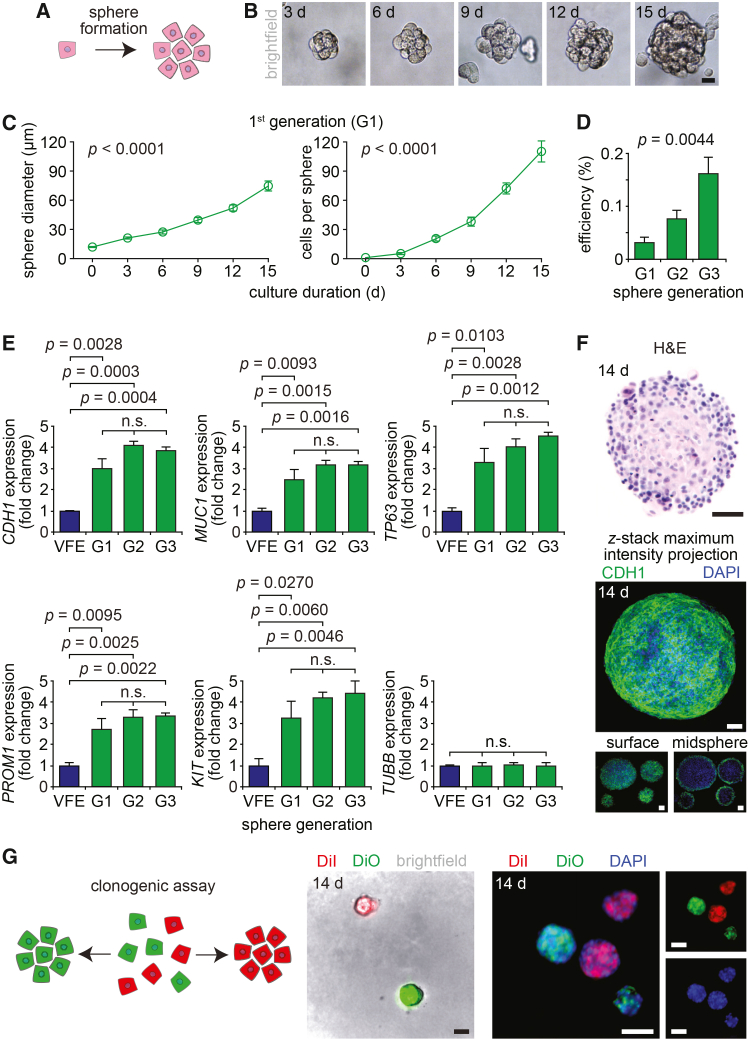
VFE progenitors generate VF clonal spheres that can be serially passaged (A) Principle of sphere formation from a single epithelial progenitor. (B) Serial bright-field images of a 1^st^ generation (G1) sphere over 15 days in anchorage-independent culture. Scale bar, 20 μm. (C) Growth curves from G1 spheres. Data are plotted as mean ± SEM (*n* = 8); *p* values were obtained using mixed-model ANOVA. (D) Sphere formation efficiency during G1–G3. Data are plotted as mean ± SEM (*n* = 6); the *p* value was obtained using mixed-model ANOVA. (E) RT-qPCR data showing *CDH1*, *MUC1*, *TP63*, *PROM1*, and *KIT* transcription in VFE spheres during G1–G3 compared with pre-G1 VFE monoculture; *TUBB* is reported as an additional reference standard. Data are presented as fold change relative to VFE (mean ± SEM; *n* = 3); *p* values were obtained using mixed-model ANOVA, with planned pairwise comparisons shown. (F) H&E- and CHD1-stained VFE spheres on day 14. Scale bars, 50 μm. The immunofluorescent images show CDH1 maximum intensity projection from a single sphere, generated from a *z* stack, as well as single *z*-plane images at the surface and midpoint of four spheres. (G) Clonogenic assay. The schematic illustrates the assay design. The fluorescent images show DiI- or DiO- (but not co-) labeled spheres in live culture and following fixation on day 14. Scale bars, 200 μm.

We next examined VFE capacity for generating 3D spheres when cultured under permissive conditions (Figure 2A). This culture approach has the potential to support primary cell expansion as well as new organotypic models of VF mucosal biology. We seeded primary VFEs at low density on ultra-low attachment plates, cultured in VFE-orientated medium, and observed gradually expanding, free-floating spheres of proliferating cells (Figures 2B and 2C). Passage culture, achieved by dissociating mature spheres and seeding the isolated cells, was examined over three generations (G1–G3). Sphere formation efficiency—defined as the percentage of seeded cells that yielded mature spheres—increased with each generation, suggesting a proportional increase in the number of progenitor cells present within the culture over time (Figure 2D). RT-qPCR showed increased transcription of all epithelial and progenitor genes of interest in spheres compared to same-donor monocultured VFEs; we observed no significant differences by sphere generation (Figure 2E). Histological assessment of mature spheres confirmed cell distribution throughout the structure; however, confocal microscopy of immunostained spheres revealed that expression of the transmembrane protein CDH1 was restricted to the sphere surface, indicating that cells within the larger sphere population exhibit depth-dependent phenotypes (Figure 2F).

To determine whether spheres are derived from individual VFE progenitors or arise via amalgamation of adjacent cells, we performed a clonogenic assay (Figure 2G). DiI- and DiO-labeled VFEs were mixed in a 1:1 ratio and cultured under sphere-formation conditions. In this assay, a clonal sphere, derived from a single labeled cell, will emit a single fluorescent signal, whereas a non-clonal sphere, derived from more than one labeled cell, will emit dual signals. Imaging of both live and fixed spheres confirmed that, at our seeding density, spheres were either DiI^+^ or DiO^+^ but not DiI^+^DiO^+^, confirming the successful generation of clonal spheres from individual VFE progenitors.

### Sphere-dissociated VFEs revert toward their original phenotype

**Figure 3:**
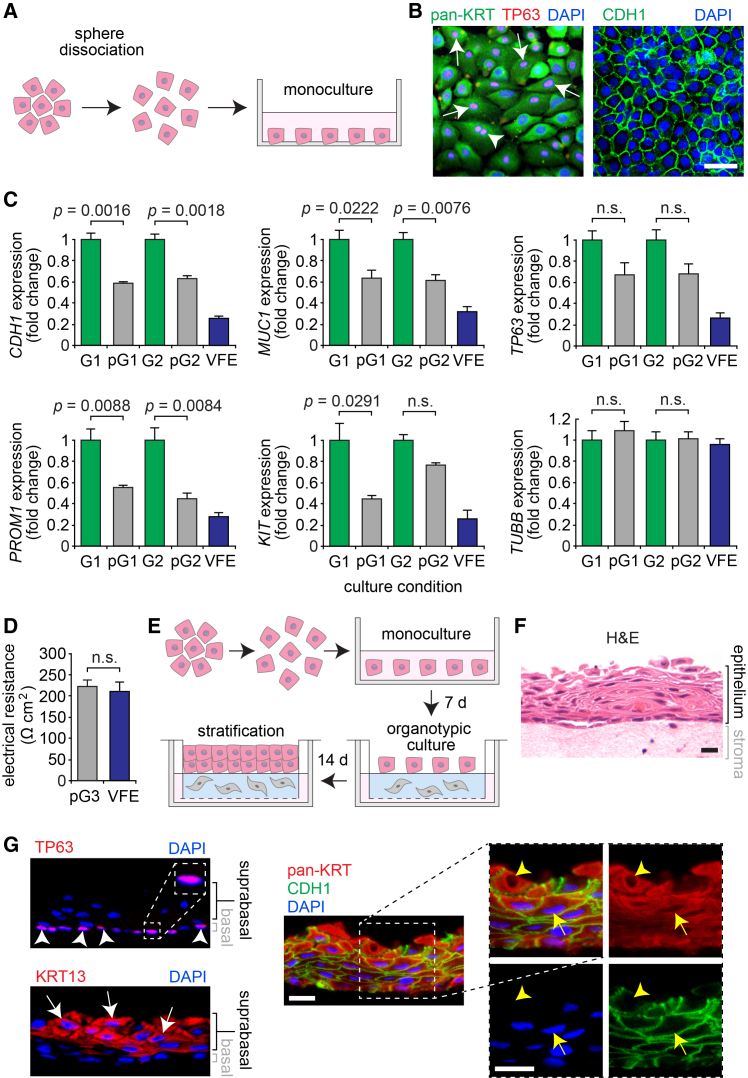
Sphere-dissociated VFEs revert toward their original phenotype in monoculture and form stratified squamous epithelium in organotypic culture (A) Principle of sphere dissociation to monoculture. (B) pan-KRT-, TP63-, and CDH1-stained VFEs in post-sphere monoculture. White arrows denote TP63^+^ nuclei; the white arrowhead denotes a TP63^+^ VFE in mitosis (either anaphase or telophase). Scale bar, 20 μm. (C) RT-qPCR data showing *CDH1*, *MUC1*, *TP63*, *PROM1*, and *KIT* transcription in pG1 and pG2 VFE monocultures compared to G1 and G2 spheres, respectively; pre-G1 VFEs are shown for comparison; *TUBB* is reported as an additional reference standard. pG1 data are presented as fold change relative to G1; pG2 data are presented as fold change relative to G2; VFE data are presented as fold change relative to the mean of G1 and G2 (mean ± SEM; *n* = 3); *p* values were obtained using mixed-model ANOVA, with planned pairwise comparisons shown. (D) Transepithelial electrical resistance. Data are plotted as mean ± SEM (*n* = 6); the *p* value was obtained using a paired *t* test. (E) Principle of organotypic culture with sphere-dissociated VFEs. (F) H&E-stained engineered VF mucosa. Scale bar, 20 μm. (G) pan-KRT-, KRT13-, TP63-, and CDH1-stained engineered VF mucosa. White arrows denote KRT13^+^ suprabasal VFEs; white arrowheads denote TP63^+^ basal VFE nuclei; yellow arrows denote a pan-KRT^+^CDH1^+^ VFE; yellow arrowheads denote a pan-KRT^+^CDH1^+^DAPI^−^ desquamating VFE at the luminal surface. Scale bars, 20 μm (10 μm, TP63 inset). Comparable immunostaining of native VF mucosa is presented in Figure S2.

To further assess the effect of sphere formation and culture on its constituent cells, we examined the phenotype of VFEs isolated from G1–G3 spheres and returned to monoculture (Figure 3A). Post-sphere cells exhibited a cuboidal morphology, pan-KRT signal localized to the cytoplasm, and CDH1 signal localized to the cell membrane; most nuclei were TP63^+^ (Figure 3B). RT-qPCR revealed that, compared with their parent spheres, post-sphere VFEs expressed fewer *CDH1*, *MUC1*, *KIT*, and *PROM* transcripts; we observed no significant difference in *TP63* expression at post-G1 and -G2 (pG1 and pG2, respectively) and *KIT* expression at pG2 (Figure 3C). Transepithelial electrical resistance at pG3 was comparable to that measured in pre-G1 VFEs (Figure 3D).

These data indicate that sphere-dissociated VFEs continue to exhibit epithelial markers and revert toward their original transcriptional and physiologic phenotype in monoculture. The sustained *TP63* expression observed at pG1 and pG2 further suggests that the post-sphere VFE population may remain enriched in progenitor cells. We next sought to determine if post-sphere VFEs retained the capacity to form stratified squamous epithelium when placed in organotypic co-culture with VFFs. Using an established method for low-passage primary cells,^13^ we seeded post-sphere monocultured VFEs on a VFF-containing collagen scaffold, transitioned VFEs to the air-liquid interface, then continued organotypic culture for 14 days (Figure 3E). The resulting engineered VF mucosa was comprised of a phenotypically appropriate ∼5-cell-thick stratified squamous epithelium and adjacent lamina propria (Figure 3F); VFEs within the epithelium were uniformly pan-KRT^+^CDH1^+^ and organized into a TP63^+^ basal layer and KRT13^+^ suprabasal layer (Figure 3G), comparable to native VF mucosa (Figure S2). These findings were replicated with pG1–pG3 cells, confirming that post-sphere VFEs retain their differentiation and stratification capacity when cultured under permissive conditions and can serve as a cell source for VF tissue engineering.

### Proteomic analysis of 3i-VFEs and spheres compared to VFEs

**Figure 4:**
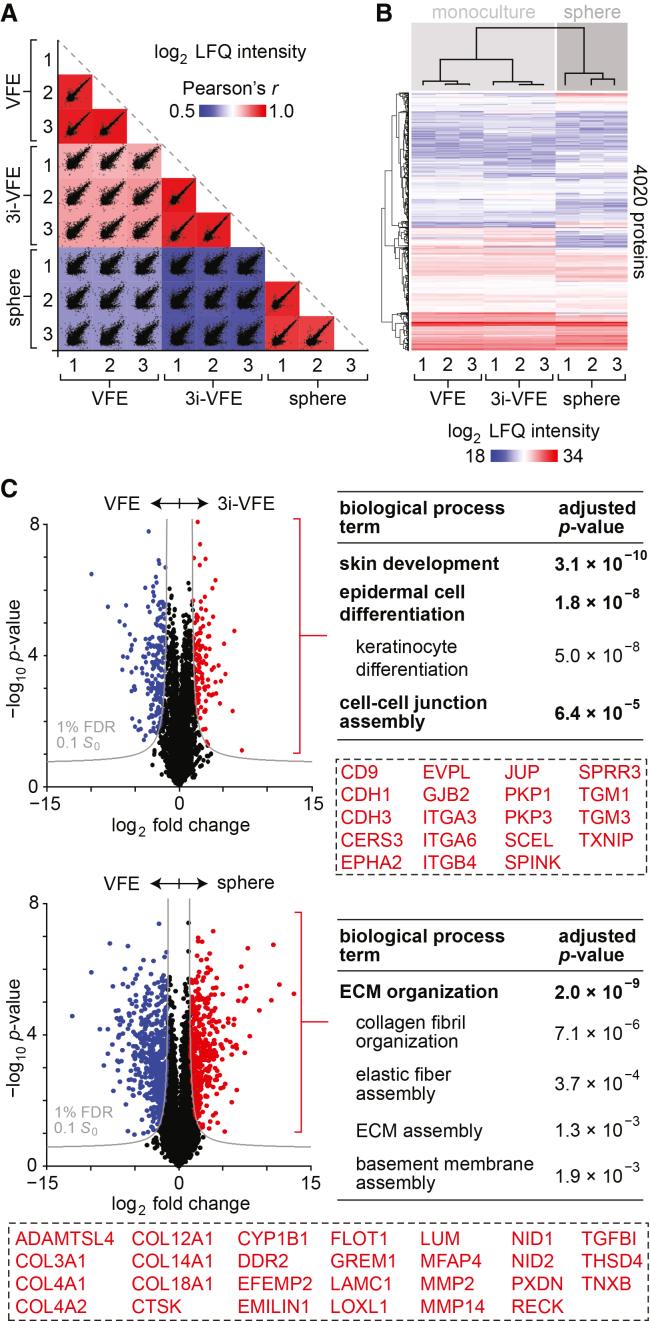
Proteomic analysis of 3i-VFEs and spheres compared to VFEs (A) Scatterplot matrix summarizing the correlation analysis of log_2_ LFQ intensities (relative protein abundances) in VFEs, 3i-VFEs, and spheres (*n* = 3 per condition). Correlation coefficients corresponding to each scatterplot (Pearson’s *r*) are represented by the heatmap overlay. (B) Hierarchical clustering analysis of log_2_ LFQ intensities across culture conditions. (C) Volcano plots summarizing differential protein abundance in VFEs compared to 3i-VFEs (upper plot) and VFEs compared to spheres (lower plot). Gray curves denote the cutoff criteria, generated in Perseus; *p* values were calculated using an unpaired *t* test. Red denotes the DA protein set with increased abundance in 3i-VFEs (*n* = 122) or spheres (*n* = 398) compared to VFEs; blue denotes the DA protein set with reduced abundance in 3i-VFEs (*n* = 198) or spheres (*n* = 509) compared to VFEs. The tables list the most significantly overrepresented Gene Ontology biological process terms in the DA protein sets with increased abundance in 3i-VFEs (upper table) and spheres (lower table), with representative terms in bold and nested terms indented; dashed rectangles highlight the specific proteins associated with enrichment of the terms in each table. Term lists were generated using Enrichr and postprocessed using REVIGO; *p* values were calculated using Fisher’s exact test with Benjamini-Hochberg adjustment. Additional enrichment data are presented in Tables S1–S4.

We conducted liquid chromatography-tandem mass spectrometry (LC-MS/MS) to assess cellular proteome changes induced by targeted inhibition of TGF-β, ROCK, and Notch, as well as by anchorage-independent sphere culture. Using a 1% false discovery rate (FDR), we identified 4,020 proteins across conditions, measured relative abundances using label-free quantification (LFQ) of spectral intensity,^34^ and evaluated group relationships via correlation and hierarchical clustering analyses. Log_2_ LFQ intensity correlations were strongest between VFEs and 3i-VFEs (*r* = 0.83–0.86), followed by spheres and VFEs (*r* = 0.64–0.65) and spheres and 3i-VFEs (*r* = 0.57–0.59) (Figure 4A). Hierarchical clustering corroborated these observations by organizing the samples into two primary clusters that corresponded to monoculture (i.e., VFE and 3i-VFE) and sphere (Figure 4B). These results demonstrated that sphere formation induces more extensive remodeling of the VFE proteome than TGF-β, ROCK, and Notch pathway inhibition.

Analysis of relative protein abundances identified 320 differentially abundant (DA) proteins in 3i-VFEs compared to VFEs, and 907 DA proteins in spheres compared to VFEs (Figure 4C). Enrichment analysis using the Gene Ontology database^35^ revealed that the DA protein set with increased abundance in 3i-VFEs (*n* = 122) was overrepresented in biological process terms associated with epithelial development and differentiation (Table S1), consistent with the increased proliferation and population expansion observed in our earlier experiments (Figure 1D). The DA protein set with increased abundance in spheres (*n* = 398) was overrepresented in terms associated with extracellular matrix (ECM) assembly and organization, fatty acid and glycan catabolism, and negative regulation of cell migration (Table S2), highlighting the structural and metabolic demands of 3D sphere formation under anchorage-independent conditions. In both pairwise comparisons with VFEs, the DA protein sets with reduced abundance in 3i-VFEs (*n* = 198) and spheres (*n* = 509) were overrepresented in an array of terms predominantly associated with cytoskeletal organization, cell motility, migration, and adhesion (Tables S3 and S4).

## Discussion

We demonstrated the effectiveness of two complementary approaches for the *in vitro* culture and expansion of primary human VFEs. Our data indicate that targeted inhibition of the TGF-β, ROCK, and Notch signaling pathways drives rapid proliferation in monoculture while maintaining core epithelial phenotype, whereas anchorage-independent culture enables population expansion via clonal sphere formation and growth in 3D context. These techniques, implemented here separately but—depending on experimental needs—usable in combination, are accessible to most suitably trained scientists without the need for specialized tools or reagents.

We developed our three-molecule inhibitor cocktail based on prior studies showing the potential of various iterations of TGF-β, ROCK, and Notch pathway inhibition, with or without feeder cell support. Chapman et al.^17^ first showed that the ROCK inhibitor Y-27632 could maintain primary human keratinocytes indefinitely on a fibroblast feeder layer, suggesting that these cells were conditionally reprogrammed into a steady proliferative state. Subsequent studies replicated this approach with prostate, mammary, and bronchial epithelial cells,^16^^,^^19^ concluding that ROCK inhibition drives proliferation by inducing telomerase and remodeling cytoskeletal dynamics. More recent work, conducted with a variety of primary epithelial cells, demonstrated that pairing Y-27632 with the TGF-β inhibitor A83-01^21^ or the Notch inhibitor DAPT^18^ allows long-term population expansion in the absence of feeder cells. A83-01 is believed to attenuate TGF-β-driven growth arrest and apoptosis,^36^ whereas DAPT promotes self-renewal and expansion of the epithelial progenitor pool.^37^ As these molecules inhibit distinct signaling pathways but exhibit synergistic effects when paired, we hypothesized that a three-component cocktail consisting of Y-27362, A83-01, and DAPT would capably support primary human VFE culture and expansion. As predicted, this approach consistently yielded successful cell isolation and rapid population expansion over serial passages.

While effective in enabling primary VFE culture and expansion, treatment with our small-molecule inhibitor cocktail presumably also impacts other biological functions related to the inhibited pathways. TGF-β, ROCK, and Notch signaling underpin an array of cellular processes, including migration, adhesion, differentiation, and various paracrine actions.^36^^,^^38^^,^^39^ We observed proteomic signatures consistent with such effects in our data, as cells in the 3i-VFE condition exhibited decreased abundance of proteins associated with cell motility, migration, and adhesion. Importantly, when implementing this strategy in an experimental setting, small-molecule inhibitors can be used for primary cell expansion and then withdrawn prior to initiating an intervention or functional assay, ensuring that the cell phenotype under investigation does not reflect unwanted off-target effects. This contrasts with immortalized epithelial cell lines derived via the transfection of viral oncogenes, such as human papillomavirus (HPV) *E6* and *E7*, or human telomerase reverse transcriptase (*TERT*), in which genomic alterations and any off-target effects are permanent.^40^^,^^41^^,^^42^^,^^43^ Such effects are a consideration when using HPV *E6*/*E7*- or *TERT*-immortalized human VFEs, both of which exhibit an epithelial phenotype but are karyotypically abnormal.^12^^,^^14^

Sphere culture offers an alternative strategy for VFE expansion alongside 3D self-assembly that reflects elements of the cell’s *in vivo* environment. We observed that VFE spheres arise from single progenitor cells and that serial passaging leads to progressive progenitor cell enrichment. VFE spheres exhibit depth-dependent CDH1 expression indicative of nascent epithelial polarity, proteome-level evidence of increased metabolic activity and ECM synthesis, and readily dissociate into phenotypically appropriate single cells as needed for monoculture, functional assays, or cryopreservation.

Driven by goals of methodological simplicity and reproducibility, we focused on sphere formation enabled by anchorage-independent culture on ultra-low attachment plates, supported by VFE-oriented medium. Sphere culture is a highly adaptable platform, however, and can be modified as needed to enrich certain cell subpopulations, promote a particular sphere morphology, create a more sophisticated (e.g., multicellular) organoid, or model a specific physiologic or disease process.^28^^,^^29^^,^^30^^,^^31^ Transitioning spheres from free-floating culture to ECM substrates, such as Matrigel, can enhance epithelial maturation. In pilot work for this study, we noted that Matrigel-cultured VFE spheres were composed of a multilayered epithelium surrounding a central lumen, as has been reported for bronchospheres,^44^ tracheospheres,^16^^,^^45^ and salispheres,^46^ which serve as phenotypically relevant models that mimic other airway and glandular epithelia. Sphere culture using growth factor-spiked basal medium can preferentially enrich stem or progenitor cells,^24^^,^^25^ whereas co-culture with stromal, endothelial, or immune cells can better represent the *in vivo* cellular milieu, enabling the study of processes such as angiogenesis, inflammation, and wound healing.^47^^,^^48^^,^^49^^,^^50^ As sphere and organoid technology continues to advance, new bioprinting and microfluidic platforms, coupled with the use of patient-derived cells and genome editing, promise to accelerate the availability of personalized prognostic and therapeutic tools. Such biomimetic advances are especially pertinent to VFEs, which exist in a phenotypically unique mechanoenvironment and have dynamic regenerative demands *in vivo*.

In summary, targeted pathway inhibition and sphere culture help overcome technical challenges that have long plagued primary human VFE cultures. These practical approaches effectively support primary cell isolation and long-term culture, enable rapid population expansion for experimental or therapeutic use, and facilitate advanced modeling of VF epithelial biology. Potential future applications of these techniques include biomechanical and disease modeling, toxicity testing, drug and biologic screening, and efforts in precision and regenerative medicine.

### Limitations of the study

First, although we isolated primary cells from eight human donors and observed minimal variation in the inhibitor responsiveness and sphere formation capacity across biological replicates, our study was not statistically powered to test for the effect of donor age or sex. Second, because our isolation protocol uses stepwise adhesion-based purification at initial cell plating and first passage to remove contaminating VFFs, we were unable to compare VFE and 3i-VFE phenotypes with same-donor unpassaged primary cells. This could be pursued in future studies using cell sorting, although such an approach might induce cell stress and further phenotypic changes. Third, while previous work using similar inhibitor cocktails has reported preservation of genomic integrity in sustained culture,^21^ 3i-VFEs may not necessarily be protected from the genomic alterations, mosaicism, genetic drift, and clonal selection that can spontaneously arise in any primary culture.^51^^,^^52^ Therefore, precautions such as screening for genomic instability via karyotyping or targeted sequencing are warranted when these cells are cultured long term.

## Resource availability

### Lead contact

Requests for further information and resources should be directed to and will be fulfilled by the lead contact, Nathan V. Welham (nvwelham@wisc.edu).

### Materials availability

This study did not generate new unique reagents.

### Data and code availability

• The raw LC-MS/MS proteomics data have been deposited to the ProteomeXchange Consortium via the PRIDE partner repository with the dataset identifier PXD064066.
• This paper does not report original code.
• Any other information required to reanalyze the data reported in this paper is available from the lead contact upon request.

## Acknowledgments

We gratefully acknowledge Erin Brooks and Jodi Corbit (Department of Pathology, University of Wisconsin-Madison) for larynx procurement; Sierra Raglan (Department of Surgery, University of Wisconsin-Madison) for histology; Lance Rodenkirch (Optical Imaging Core, University of Wisconsin-Madison) for confocal microscopy consultation; Melinda Herbath and Zsuzsanna Fabry (Department of Pathology, University of Wisconsin-Madison) for assistance with the transepithelial electrical resistance assay; and Glen Leverson (Department of Surgery, University of Wisconsin-Madison) for statistical consultation. This work was supported by grants R21 DC017836 (X.S. and N.V.W.), R01 DC004428 (N.V.W.), R01 DC010777 (N.V.W.), and R01 DC019357 (N.V.W.) from the 10.13039/100000055National Institute on Deafness and Other Communication Disorders; grants R01 AG052324 (L.L.) and R01 AG078794 (L.L.) from the 10.13039/100000049National Institute on Aging; and grant R01 DK071801 (L.L.) from the 10.13039/100000062National Institute of Diabetes and Digestive and Kidney Diseases. L.L. additionally wishes to acknowledge shared instrumentation grants S10 RR029531, S10 OD028473, and S10 OD025084 for supporting the acquisition of mass spectrometers. Flow cytometry was performed in the Flow Cytometry Laboratory of the 10.13039/100007923University of Wisconsin Carbone Cancer Center, which is supported by grant P30 CA014520 from the 10.13039/100000054National Cancer Institute.

## Author contributions

X.S. and N.V.W. designed the study; X.S. and R.S. conducted cell culture experiments; X.S. performed RT-qPCR, flow cytometry, immunostaining, and microscopy; H.L. and H.Z. collected and analyzed LC-MS/MS data with guidance from L.L.; X.S. and N.V.W. wrote the manuscript; and all authors reviewed and approved the final version.

## Declaration of interests

The authors declare no competing interests.

## STAR★Methods

### Key resources table

**Table:** 

REAGENT or RESOURCE	SOURCE	IDENTIFIER
**Antibodies**		

Mouse monoclonal anti-KRT14, FITC conjugated, clone LL002 (1:10 dilution)	Abcam	Cat# ab77684; RRID: AB_2265437
Mouse monoclonal anti-KRT19, PerCP conjugated, clone RCK108 (1:10 dilution)	Santa Cruz Biotechnology	Cat# sc-53003; RRID: AB_629839
Mouse monoclonal anti-CD90, PE-Cy7 conjugated, clone 5E10 (1:20 dilution)	BD Biosciences	Cat# 561558; RRID: AB_10714644
Mouse monoclonal anti-MUC1/CD227, PE conjugated, clone 16A (1:20 dilution)	BioLegend	Cat# 355603; RRID: AB_2561643
Rabbit monoclonal anti-CDH1, clone EP700Y (1:400 dilution)	Abcam	Cat# ab40772; RRID: AB_731493
Mouse monoclonal anti-pan-KRT, clone C11 (1:400 dilution)	Cell Signaling Technology	Cat# 4545; RRID: AB_490860
Mouse monoclonal anti-KRT13, clone A-3 (1:50 dilution)	Santa Cruz Biotechnology	Cat# sc-390982
Mouse monoclonal anti-TP63, clone D-9 (1:50 dilution)	Santa Cruz Biotechnology	Cat# sc-25268; RRID: AB_628092
Donkey polyclonal anti-mouse IgG, Alexa Fluor 488 conjugated (1:200 dilution)	Thermo Fisher Scientific	Cat# A-21202; RRID: AB_141607
Donkey polyclonal anti-mouse IgG, Alexa Fluor 594 conjugated (1:200 dilution)	Thermo Fisher Scientific	Cat# A-21203; RRID: AB_2535789
Donkey polyclonal anti-rabbit IgG, Alexa Fluor 488 conjugated (1:200 dilution)	Thermo Fisher Scientific	Cat# A-21206; RRID: AB_2535792
Mouse monoclonal IgG1 κ, clone MOPC-21, PE conjugated (1:20 dilution)	BioLegend	Cat# 981804; RRID: AB_3076354
Mouse monoclonal IgG1 κ, clone MOPC-21, PE-Cy7 conjugated (1:20 dilution)	BD Biosciences	Cat# 557872; RRID: AB_396914
Mouse monoclonal IgG1 κ, clone MOPC-21, PerCP conjugated (1:5 dilution)	BD Biosciences	Cat# 559425; RRID: AB_397240
Mouse monoclonal IgG3, clone PPV-07, FITC conjugated (1:5 dilution)	Abcam	Cat# ab91539
Mouse monoclonal IgG1 κ, clone B11/6 (1:100 dilution)	Abcam	Cat# ab91353; RRID: AB_2811128
Mouse polyclonal IgG (1:40 dilution)	Santa Cruz Biotechnology	Cat# sc-2025; RRID: AB_737182
Mouse polyclonal IgG2a (1:5 dilution)	Santa Cruz Biotechnology	Cat# sc-3878; RRID: AB_737242
Rabbit monoclonal IgG, clone EPR25A (1:200 dilution)	Abcam	Cat# ab172730; RRID: AB_2687931

**Biological samples**		

Cadaveric human larynges	Department of Pathology, University of Wisconsin-Madison	N/A

**Chemicals, peptides, and recombinant proteins**		

A-83-01	Selleck Chemicals	Cat# S7692; CAS: 909910-43-6
Y-27632	Selleck Chemicals	Cat# S1049; CAS: 129830-38-2
DAPT	Selleck Chemicals	Cat# S2215; CAS: 208255-80-5
Type I collagenase	Sigma Aldrich	Cat# C0130; CAS: 9001-12-1
Type I collagen, rat tail	Corning Life Sciences	Cat# 354236; CAS: 9007-34-5
Fibronectin, human	Corning Life Sciences	Cat# 354008; CAS: 86088-83-7
Bovine pituitary extract	Sigma Aldrich	Cat# 02-104
Epidermal growth factor	Sigma Aldrich	Cat# E5036; CAS: 62253-63-8
Epinephrine	Sigma Aldrich	Cat# E4250; CAS: 51-43-4
Insulin, human recombinant	Sigma Aldrich	Cat# 91077C; CAS: 11061-68-0
Transferrin, human	Sigma Aldrich	Cat# T8158; CAS: 11096-37-0
Triiodo-L-thyronine	Sigma Aldrich	Cat# T5516; CAS: 55-06-1
Hydrocortisone	Sigma Aldrich	Cat# H0888; CAS: 50-23-7
Retinoic acid	Sigma Aldrich	Cat# R2625; CAS: 302-79-4
Citrate buffer	Sigma Aldrich	Cat# C9999
Donkey serum	Sigma-Aldrich	Cat# D9663
HBSS	Lonza Bioscience	Cat# 04-315Q
Trypan blue	Thermo Fisher Scientific	Cat# 15250061; CAS: 72-57-1
FBS	Thermo Fisher Scientific	Cat# 16000044
Antibiotic-antimycotic solution	Thermo Fisher Scientific	Cat# 15240062; CAS: 61-33-6 (penicillin G), CAS: 3810-74-0 (streptomycin), CAS: 1397-89-3 (amphotericin B)
PBS	Thermo Fisher Scientific	Cat# 10010049
DNase I	Thermo Fisher Scientific	Cat# J61061.FPL; CAS:9003-98-9
DMEM	Thermo Fisher Scientific	Cat# 11965118
DMEM/Ham’s F-12	Thermo Fisher Scientific	Cat# 11320082
Trypsin-EDTA	Thermo Fisher Scientific	Cat# 25300054
DMSO	Thermo Fisher Scientific	Cat# 036480.AP; CAS: 67-68-5
BSA	Sigma Aldrich	Cat# A7906; CAS: 9048-46-8
DiI	Thermo Fisher Scientific	Cat# V22885; CAS: 41085-99-8
DiO	Thermo Fisher Scientific	Cat# V22886; CAS: 2171344-23-1
PowerUp SYBR Green master mix	Applied Biosystems	Cat# A25742
HEPES	Thermo Fisher Scientific	Cat# J63002.AE; CAS: 7365-45-9
PFA	Thermo Fisher Scientific	Cat# J61899.AK; CAS: 30525-89-4
Triton X-100	Thermo Fisher Scientific	Cat# 85111; CAS: 9036-19-5
DAPI	Thermo Fisher Scientific	Cat# D1306; CAS: 28718-90-3
Urea	Thermo Fisher Scientific	Cat# J75826.A1; CAS: 57-13-6
Tris-HCl	Thermo Fisher Scientific	Cat# J22638.AE; CAS: 1185-53-1
Dithiothreitol	Thermo Fisher Scientific	Cat# 15508013; CAS: 3483-12-3
Iodoacetamide	Thermo Fisher Scientific	Cat# 122270050; CAS: 144-48-9
Trifluoroacetic acid	Thermo Fisher Scientific	Cat# 432295000; CAS: 76-05-1
Formic acid	Thermo Fisher Scientific	Cat# 270480250; CAS: 64-18-6
Acetonitrile	Thermo Fisher Scientific	Cat# 047138.M1; CAS: 75-05-8
Trypsin/Lys-C	Promega	Cat# V5073; CAS: 9002−07−7 (trypsin), CAS: 72561-05-8 (Lys-C)

**Critical commercial assays**		

RNeasy kit	Qiagen	Cat# 74104
High-capacity cDNA reverse transcription kit	Applied Biosciences	Cat# 4374966
FIX & PERM cell permeabilization kit	Thermo Fisher Scientific	Cat# GAS004
Pierce bicinchoninic acid protein assay kit	Thermo Fisher Scientific	Cat# 23227
Pierce quantitative colorimetric peptide assay kit	Thermo Fisher Scientific	Cat# 23275

**Deposited data**		

Raw mass spectrometry data	This paper	PRIDE repository (http://www.ebi.ac.uk/pride/), dataset identifier PXD064066; RRID: SCR_003411
*Homo sapiens* reference proteome (downloaded August 2023)	UniProt	http://www.uniprot.org; RRID: SCR_002380

**Oligonucleotides**		

Primers for *TP63*, *PROM1*, *KIT*, *CDH1*, *MUC1*, *TUBB*, and *SDHA*, see Table S6	This paper	N/A

**Software and algorithms**		

MaxQuant 2.0.3.0	Jürgen Cox, Max Planck Institute of Biochemistry	http://maxquant.org/; RRID: SCR_014485
Perseus 2.0.11	Jürgen Cox, Max Planck Institute of Biochemistry	http://maxquant.org/perseus/; RRID: SCR_015753
Enrichr (accessed February 2024)	Avi Ma’ayan, Icahn School of Medicine at Mount Sinai	http://maayanlab.cloud/enrichr/; RRID: SCR_001575
REVIGO (accessed March 2024)	Fran Supek, Ruđer Bošković Institute	http://revigo.irb.hr/; RRID: SCR_005825
FlowJo 10.6.1	BD Biosciences	http://www.flowjo.com/; RRID: SCR_008520
SAS 9.2	SAS Institute	http://www.sas.com/; RRID: SCR_008567

**Other**		

100-mm-diameter ultra-low attachment plates	Corning Life Sciences	Cat# 4615
Falcon 0.4-μm-pore-size, 24-well culture inserts	Corning Life Sciences	Cat# 353095
Nunc 0.4-μm-pore-size, 12-well culture inserts	Thermo Fisher Scientific	Cat# 140652
Concavity slides	Carolina Biological Supply	Cat# 632200
Vectashield	Vector Laboratories	Cat# H-1700-2
TissueGel	Morphisto	Cat# 10059.VE012

### Experimental model and study participant details

#### Human cadaveric tissue

We procured nine human larynges (*n* = 5 male, *n* = 4 female; age range, 18–83 years) from cadavers at autopsy with approval of the University of Wisconsin-Madison Health Sciences Institutional Review Board. Individual demographic and clinical data are reported in Table S5. Eight specimens were used for primary cell isolation and culture; one specimen was used for histology. Preliminary observations showed no sex- or age-related effect on cell proliferation or sphere formation capacity and so these variables were not considered in later experiments.

### Method details

#### Primary cell isolation and culture

Larynges were transected at midline in the sagittal plane and visually inspected to rule out obvious pathology; each VF mucosa was microdissected from its underlying thyroarytenoid muscle. Samples were minced with scalpels and then incubated in PBS containing 5% FBS (Thermo Fisher), 7.5 mg/mL type I collagenase (Sigma Aldrich), and 0.2 mg/mL DNase I (Thermo Fisher) at 37°C for 1–3 h. Following a triple wash with PBS, the released cells were passed through a 40-μm filter (BD Biosciences), resuspended in VFF-orientated medium (DMEM containing 10% FBS and 100 U/mL antibiotic-mycotic solution; Thermo Fisher), and incubated at 37°C in 5% CO_2_. After 1–2 h, non-adherent cells were collected, washed with PBS, and resuspended in VFE-orientated medium [DMEM/Ham’s F-12 containing 1% FBS and 100 U/mL antibiotic-antimycotic solution (Thermo Fisher), supplemented with 15 μg/mL bovine pituitary extract, 10 ng/mL epidermal growth factor, 0.5 μg/mL epinephrine, 5 μg/mL insulin, 10 μg/mL transferrin, 10 ng/mL triiodo-L-thyronine, 0.5 μg/mL hydrocortisone, 0.1 ng/mL retinoic acid, 1.5 μg/mL BSA; Sigma Aldrich] on type I collagen- and fibronectin-coated plates (Corning). Adherent cells were maintained in VFF-orientated medium on uncoated plates. All cells were cultured at 37°C in 5% CO_2_ with medium change every 48 h; cells were passaged when 70–80% confluent. Unless noted otherwise (e.g., doubling times in Figure 1D), we conducted experiments with passage 2–4 cells.

The above isolation protocol is based on prior work showing that suspended VFF attach more readily to a culture surface than VFE; once attached, however, VFE require a more aggressive dissociation strategy than VFF to successfully release.^13^ We therefore used stepwise trypsinization to further purify the VFE subpopulation at first passage, as follows. Cells in VFE-orientated medium were washed once with PBS, incubated with 0.05% trypsin-EDTA (Thermo Fisher) at 37°C for 1-3 min to detach contaminating VFF, then incubated with 0.25% trypsin-EDTA at 37°C for 3-5 min to detach remaining VFE for passage culture.

#### Targeted pathway inhibition

We piloted the individual and combined effect of each candidate small-molecule inhibitor on VFE proliferation as follows. VFE were seeded in 24-well plates (Corning) at a density of 5 × 10^4^ cells per well, cultured in VFE-orientated medium until ∼30% confluent, serum starved for 12 h, then incubated with various combinations of 1 μM TGF-β inhibitor A-83-01, 10 μM ROCK inhibitor Y-27632, and 5 μM Notch inhibitor DAPT (Selleck Chemicals), each prepared from a 1,000× stock solution in DMSO (Thermo Fisher). Dosing was based on published literature^18^^,^^21^; we conducted additional dose-response testing for DAPT (0–15 μM) due to a report of dose-dependent cytotoxicity in mouse tracheal epithelial cells.^53^ Control cells were maintained in VFE-orientated medium with DMSO vehicle. Cells were harvested at 9 days; counts were performed in technical triplicate using a hemocytometer.

For all subsequent experiments, cells assigned to the 3i-VFE condition were cultured in VFE-orientated medium containing a three-molecule cocktail of 1 μM A-83-01, 10 μM Y-27632, and 5 μM DAPT in DMSO. Chemical inhibition began at the time of cell plating; cells assigned to the VFE (control) condition were cultured with DMSO vehicle.

#### Sphere culture

Monocultured VFE were harvested, washed with PBS, resuspended in VFE-orientated medium, and plated on 100-mm-diameter ultra-low attachment plates (Corning) at a density of 0.5–5 × 10^4^ cells/mL. Cells (and nascent spheres) were maintained in VFE-orientated medium and cultured at 37°C in 5% CO_2_ with medium change every 72 h.

Spheres intended for passage culture were harvested at 15 days, centrifuged at 400 × *g* for 5 min, dissociated with ice-cold 0.05% trypsin-EDTA for 20 min, and agitated with a polished glass pipette. Single cells were separated from sphere clumps using a 40-μm filter (BD Biosciences), stained with trypan blue (Thermo Fisher), and counted. Viable cells were resuspended in VFE-orientated medium and plated for next-generation sphere or monolayer culture.

#### Organotypic culture

We engineered VF mucosae using organotypic culture as previously described.^13^ Purified type I collagen (3.5 mg/mL; Corning) was seeded with 4 × 10^5^ VFF/mL, placed in the apical chamber of a 0.4-μm-pore-size, 24-well culture insert (Falcon; Corning), then polymerized. VFF were cultured in VFF-orientated medium (added to both apical and basolateral chambers) for 24 h. Next, VFE were seeded on the scaffold surface, VFE-orientated medium was added to the apical chamber, and a 1:1 ratio of VFF- and VFE-orientated medium was added to the basolateral chamber. Media were changed every 24 h; at 72 h, the VFE-orientated medium was aspirated from the apical chamber and organotypic culture continued with VFE at the air-liquid interface for 14 days.

#### Proliferation assays

Monocultured VFE were split and 5,000 cells per well were plated on 12-well plates (Corning). Cells were cultured with (3i-VFE condition) or without (VFE condition) pathway inhibitors, then harvested at the indicated timepoints (Figure 1D). Counts were performed in technical triplicate using a hemocytometer. Population doubling times were calculated by plating 10^5^ cells per well in 6-well plates (Corning), culturing for 144 h (6 days), then harvesting and counting in technical triplicate. Sphere diameters were measured via serial imaging at the indicated timepoints (Figure 2C) using an inverted microscope (Ti-S/L100; Nikon). Within-sphere cell counts were performed following sphere dissociation to single cells at the same timepoints, in technical triplicate using a hemocytometer.

#### Clonogenic assay

Monocultured VFE were harvested and 10^6^ cells per labeling condition were incubated with 5 μM DiI or DiO (Invitrogen V22885, V22886; Thermo Fisher) in HBSS (Lonza) at 37°C for 20 min. Cells were washed with pre-warmed VFE-oriented medium and labeling was confirmed with a fluorescent microscope (Ti-S/L100; Nikon). DiI- and DiO-labeled cells were mixed at a 1:1 ratio, cultured under sphere-formation conditions, and imaged at 14 days.

#### RT-qPCR

Total RNA was extracted from monocultured VFE (2 × 10^5^ per replicate) and spheres (∼200 per replicate) using a Qiagen RNeasy kit according to the manufacturer’s protocol. RNA yield was quantified using a NanoDrop spectrophotometer (Thermo Fisher); 1 μg total RNA from each sample was then reverse transcribed using a High-Capacity cDNA Reverse Transcription kit (Applied Biosystems) according to the manufacturer’s protocol.

We performed RT-qPCR on an Applied Biosystems 7500 Fast Real-Time PCR system. Each 25 μL reaction contained 1 μL cDNA template, 2 μL primers (final concentration, 200 nM; Table S6), 9.5 μL nuclease-free water, and 12.5 μL PowerUp SYBR Green master mix (Applied Biosystems). The cycling conditions were as follows: activation at 95°C for 10 min, followed by 40 cycles of 94°C for 15 s and 60°C for 30 s. Reactions were performed in technical triplicate; amplification specificity was confirmed by the presence of a single distinct melting curve; negative controls, in which reverse transcription was performed without RNA template or RT-qPCR was performed without cDNA template, showed no target amplification.

#### Flow cytometry

Cells were washed and suspended in staining buffer (PBS containing 5% BSA, 5% FBS, and 10 mM HEPES). For surface marker staining, cells were incubated with fluorochrome-conjugated antibodies (key resources table) at room temperature (RT) for 30 min in the dark. For intracellular staining, cells were first fixed and permeabilized using a commercial kit (Invitrogen FIX & PERM; Thermo Fisher) according to the manufacturer’s protocol, then incubated with fluorochrome-conjugated antibodies against the intracellular targets of interest (key resources table) at RT for 60 min in the dark. Finally, cells were washed, pelleted, and resuspended in staining buffer for flow cytometry. Samples were run on a FACSCalibur instrument (BD Biosciences).

#### Transepithelial electrical resistance

VFE were plated on type I collagen-coated, 0.4-μm-pore-size, 12-well inserts (Nunc; Thermo Fisher) at a density of 10^5^ cells per well and cultured for 14 days. Electrical resistance measurements were performed in technical duplicate using a Millicell ERS-2 volt ohm meter (Millipore), according to the manufacturer’s instructions. Background resistance data were collected from collagen-coated inserts containing medium but no cells.

#### Histology, ICC and IHC

Monolayered VFE were cultured on type I collagen-coated chamber slides for 7–10 days, washed with PBS, fixed with 4% PFA for 10–15 min, permeabilized with 0.1% Triton X-100 in PBS for 5 min, and blocked with 5% BSA and 5% donkey serum (Sigma Aldrich) in PBS for 2 h (all at RT). Fixed cells were incubated with primary antibodies (key resources table) at 4°C overnight, followed by appropriate fluorophore-conjugated secondary antibodies (key resources table) at RT for 1 h in the dark. Cells were counterstained with 300 nM DAPI (Sigma-Aldrich) at RT for 1–5 min, covered with Vectashield antifade mounting medium (Vector Laboratories), and coverslipped.

VFE spheres were fixed with 4% PFA for 4 h, permeabilized with 0.1% Triton X-100 in PBS for 1 h, and blocked with 5% BSA and 5% donkey serum in PBS for 6 h (all at 4°C). Fixed spheres were incubated with a rabbit anti-human CDH1 primary antibody (key resources table) at 4°C for 72 h, followed by a fluorophore-conjugated donkey anti-rabbit IgG secondary antibody (key resources table) at 4°C for 12 h in the dark. We performed thorough washing between each incubation step. Spheres were counterstained with 300 nM DAPI at 4°C for 30 min, then transferred to concavity slides (Carolina Biological), mounted with Vectashield, and coverslipped.

A subset of spheres, all engineered VF mucosae, and one native VF mucosa were washed with PBS, fixed with 4% PFA for 30 min, and paraffin embedded [spheres were suspended in TissueGel (Morphisto) after fixation and prior to paraffin embedding]. Five-μm thick paraffin sections were prepared and processed for H&E- and immunostaining. Sections intended for immunostaining underwent antigen retrieval with 10 mM citrate buffer (pH 6.0; Sigma Aldrich) at 95°C for 45–60 min and blocking with 5% BSA and 5% donkey serum in PBS at RT for 1 h. Sections were incubated with primary antibodies (key resources table) at 4°C for 12 h, followed by appropriate fluorophore-conjugated secondary antibodies (key resources table) at RT for 1 h in the dark. Sections were counterstained with 300 nM DAPI at RT for 1–5 min, mounted with Vectashield, and coverslipped.

Microscopy was performed using a Nikon Eclipse E600 microscope connected to an Olympus DP73 digital camera and a Nikon Ti-S/L100 inverted microscope connected to DS-Qi2 digital camera; spheres were additionally imaged using a Nikon Yokogawa CSU-W1 spinning disk confocal microscope (30 images per sphere were acquired in the *z*-plane at a field depth of 3 μm); confocal images were postprocessed using Nikon NIS-Elements software. Samples were imaged with consistent exposure settings; negative control sections stained with an appropriate isotype control (key resources table), or with no primary or secondary antibody incubation step, showed no signal.

#### LC-MS/MS

Proteins were extracted from each sample by first adding 150 μL of 8 M urea and 50 mM Tris-HCl (pH 8), sonicating (alternating 15 s on/off cycles) for 1 min, and then centrifuging at 14,000 × *g* for 15 min at 4°C to remove cellular debris. Protein concentration was determined using a bicinchoninic acid assay kit (Pierce; Thermo Fisher). Proteins were reduced to 5 mM dithiothreitol (DTT) for 30 min at 37°C, then alkylated to 15 mM iodoacetamide for 45 min at RT in the dark; the reaction was quenched by adding DTT to 5 mM for 10 min at RT. The protein mixture was diluted with 50 mM Tris-HCl (pH 8) to reduce the urea concentration to ≤1 M, and then digested with Trypsin/Lys-C (Promega) [50:1 (w/w) protein/enzyme ratio] at 37°C overnight. Digestion was quenched by acidifying the sample with 10% trifluoroacetic acid to a final pH < 3. The digested sample was desalted using a Sep Pak C18 1 cc Vac cartridge (Waters). Peptides were first eluted with 0.1% formic acid (FA) in 50% acetonitrile (ACN) and then with 0.1% FA in 80% ACN. The eluate was evaporated to dryness in a vacuum centrifuge and peptide concentration was determined using a quantitative colorimetric peptide assay (Pierce; Thermo Fisher).

LC-MS/MS was performed using three technical replicates per biological replicate. Each sample was dissolved in 0.1% FA; 1 μg of the digested peptides was injected into a Vanquish Neo UHPLC system coupled to an Orbitrap Exploris 480 mass spectrometer (Thermo Fisher). Peptides were separated using an in-house, 15-cm-long, 75-μm-inner-diameter microcapillary column packed with ethylene bridged hybrid C18 particles (1.7 μm, 130 Å; Waters). Mobile phase A consisted of H_2_O with 0.1% FA; mobile phase B consisted of 80% ACN with 0.1% FA. Separation was achieved using a 3.0–37.5% mobile phase B gradient over 102 min at a flow rate of 300 nL/min.

A full mass scan (*m/z* 350–1200) was performed using the Orbitrap at a resolution of 60,000 in data-dependent acquisition mode. The normalized automatic gain control target was set to 300%, the maximum injection time mode was set to Auto, and the exclusion duration was 30 s. Precursors were fragmented by high-energy collisional dissociation with a collision energy of 30%, a resolution of 15,000, an isolation width of *m/z* 2.0, a lower mass limit of *m/z* 120, and a maximum injection time of 40 ms.

### Quantification and statistical analyses

#### Growth kinetics

All count-based data were measured in technical triplicate. Population doubling time was calculated as

$$T_{d}=\frac{t\cdot \ln\left(2\right)}{\ln\left(N_{t}/N_{0}\right)}$$

where *T*_*d*_ is the population doubling time, *t* is the time in culture, *N*_0_ is the number of cells plated at time 0, and *N*_*t*_ is the number of cells harvested at time *t*.

Cross-sectional sphere diameters were measured using ImageJ.^54^ Within-sphere cell counts were performed by dissociating 30 spheres per replicate, then dividing the total count by 30 to obtain a mean cell-per-sphere value for that replicate. Sphere formation efficiency was calculated by dividing the total number of spheres identified at 15 days by the number of cells plated at time 0, then converting to a percentage.

#### mRNA expression

All RT-qPCR reactions were performed in technical triplicate. Relative mRNA expression was calculated using the 2^–ΔΔCT^ method^55^; values were normalized against reference gene *SDHA*. The β-tubulin class I gene *TUBB* was employed as an additional reference standard.

#### Flow cytometry

Flow cytometry data were analyzed using FlowJo 10.6.1 (BD Biosciences). We used both unstained and isotype (key resources table) controls for population gating.

#### Transepithelial electrical resistance

All electrical resistance measurements were performed in technical duplicate. Transepithelial electrical resistance was calculated by subtracting background resistance (measured in collagen-coated inserts containing medium but no cells) from total resistance and then multiplying by the surface area of the 12-well insert (1.13 cm^2^).

#### Proteomics

Protein identification and label-free quantification (LFQ) were performed using MaxQuant 2.0.3.0.^56^ The raw mass data were searched against the UniProt *Homo sapiens* reviewed database (downloaded August 2023) with trypsin/P selected as the digestion enzyme. Two missed cleavages were allowed, a minimum of two unique peptides per protein identification was required, and the results were filtered using a 1% peptide FDR. The first search peptide tolerance was set to 20 ppm, while the main search peptide tolerance was set to 4.5 ppm. Variable modifications included methionine oxidation (+15.995 Da) and protein N-terminal acetylation (+42.010 Da), while static carbamidomethylation of cysteines (+57.021 Da) was applied. Match between runs was enabled; all other parameters were set to default.

#### Statistical analyses

Data presentation formats, sample sizes, and analysis methods are summarized in each figure legend.

Non-proteomic data were analyzed using SAS 9.2 (SAS Institute). Data were first evaluated for normality and equality of variance using visual inspection of raw data plots and folded *F* tests; all data met the necessary assumptions. Flow cytometry, monoculture RT-qPCR, and transepithelial electrical resistance data were analyzed using paired or unpaired *t* tests, as appropriate. Cell proliferation, sphere growth, and sphere (including post-sphere monoculture) RT-qPCR data were analyzed using mixed-model ANOVAs, with primary cell donor as a random effect and each independent variable of interest as a fixed effect. In all ANOVA models, if the *F* test revealed a significant difference, planned pairwise comparisons were performed using Fisher’s protected least significant difference method. A type I error rate of 0.05 was used; all *p-*values were two-sided.

Quantitative proteomic data (log_2_ LFQ intensity) were analyzed in Perseus 2.0.11^57^ using a 1% FDR and artificial within-groups variance (*S*_0_) of 1.0. Additional analyses were conducted in Perseus using hierarchical clustering and calculation of Pearson’s *r*. Enrichment analysis of DA proteins was performed using the Gene Ontology database^35^ and Enrichr algorithm^58^ (based on Fisher’s exact test with Benjamini-Hochberg adjustment), a preadjustment type I error rate of 0.01, and requirement of at least four proteins per ontology term. Enrichr output was postprocessed using the REVIGO semantic similarity algorithm.^59^

## Supplemental information

Document S1. Figures S1 and S2 and Tables S1–S6

Document S2. Article plus supplemental information
