# Supplementary material for: Rapid expansion of primary human vocal fold epithelial cells via targeted pathway inhibition and anchorage-independent sphere culture
Source: Cell Rep Methods. 2026 Mar 6;6(3):101310. doi: 10.1016/j.crmeth.2026.101310 (PMC13030965; doi:10.1016/j.crmeth.2026.101310)
Supplement: Document S1. Figures S1 and S2 and Tables S1–S6 [file mmc1.pdf]

Cell Reports Methods, Volume *6*

Supplemental information

Rapid expansion of primary human vocal fold epithelial cells via targeted pathway inhibition and anchorage-independent sphere culture

Xudong Shi, Ryo Suzuki, Haiyan Lu, Hua Zhang, Lingjun Li, and Nathan V. Welham

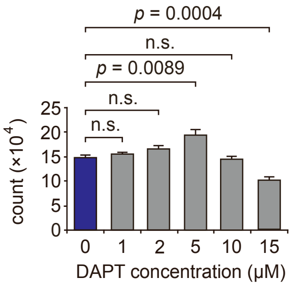

Figure S1. Effect of DAPT concentration on VFE proliferation, related to Figure 1. Cells (5×10^4) were incubated with 0-15 μM DAPT; counts were performed at 9 d; data are plotted as means ± SEM (n = 6); p-values were obtained using mixed-model ANOVA with planned pairwise comparisons shown.

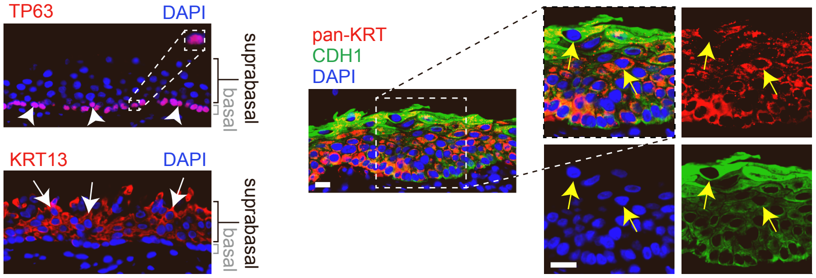

Figure S2. Pan-KRT-, KRT13-, TP63-, and CDH1-stained native human VF mucosa, related to Figure 3. White arrows denote KRT13$^+$ suprabasal VFEs; white arrowheads denote TP63$^+$ basal VFE nuclei; yellow arrows denote pan-KRT$^+$CDH1$^+$ VFE. Scale bars, 20 μm (10 μm, TP63 inset).

Table S1. Gene ontology biological process terms overrepresented in the DA protein set with increased abundance in 3i-VFE compared to VFE, related to Figure 4. Terms were identified using the Enrichr algorithm based on a Benjamini Hochberg-adjusted *p*-value < 0.01 and minimum of 4 protein identifications per pathway, then postprocessed for redundancy using the REVIGO semantic similarity algorithm. Data are presented in order of ascending adjusted *p*-value of the representative term (in bold) for each cluster; nested terms are in gray and italicized. The top 3 clusters are presented in Figure 4C.

Term	Description	Proteins	Adjusted *p*-value
**GO:0043588**	**skin development**	TGM1; CERS3; SPRR3; SCEL; ITGB4; ITGA3; TXNIP; ITGA6; EVPL; TGM3; EPHA2	3.14E-10
**GO:0009913**	**epidermal cell differentiation**	TGM1; CERS3; SPRR3; SCEL; SPINK5; TXNIP; EVPL; TGM3; EPHA2	1.83E-08
GO:0030216	*keratinocyte differentiation*	TGM1; CERS3; SPRR3; SCEL; TXNIP; EVPL; TGM3; EPHA2	5.02E-08
**GO:0007043**	**cell-cell junction assembly**	GJB2; CDH3; JUP; CDH1; PKP1; CD9; PKP3	6.35E-05
**GO:0010544**	**negative regulation of platelet activation**	PDGFRA; CEACAM1; CD9; APOE	3.41E-04
**GO:0008544**	**epidermis development**	CERS3; ALDH3A2; SPRR3; SCEL; SPINK5; EVPL	0.0019
**GO:0016101**	**diterpenoid metabolic process**	ALDH3A2; AKR1B10; ADH1B; ADH7	0.0030

Table S2. Gene ontology biological process terms overrepresented in the DA protein set with increased abundance in sphere compared to VFE, related to Figure 4. Terms were identified using the Enrichr algorithm based on a Benjamini Hochberg-adjusted *p*-value < 0.01 and minimum of 4 protein identifications per pathway, then postprocessed for redundancy using the REVIGO semantic similarity algorithm. Data are presented in order of ascending adjusted *p*-value of the representative term (in bold) for each cluster; nested terms are in gray and italicized. The top cluster is presented in Figure 4C.

Term	Description	Proteins	Adjusted *p*-value
**GO:0030198**	**extracellular matrix organization**	COL18A1; COL14A1; LUM; MMP2; COL12A1; LAMC1; NID1; NID2; LOXL1; THSD4; GREM1; MMP14; COL3A1; COL4A2; COL4A1; ADAMTSL4; CTSK; PXDN; FLOT1; CYP1B1; TGFBI; RECK; DDR2	2.01E-09
GO:0030199	*collagen fibril organization*	GREM1; COL18A1; COL3A1; COL14A1; LUM; COL12A1; PXDN; CYP1B1; LOXL1; DDR2	7.80E-06
GO:0048251	*elastic fiber assembly*	MFAP4; EFEMP2; TNXB; EMILIN1	3.71E-04
GO:0085029	*extracellular matrix assembly*	MFAP4; EFEMP2; TNXB; PXDN; EMILIN1	0.0013
GO:0071711	*basement membrane organization*	COL4A1; PXDN; NID1; NID2	0.0019
**GO:0009062**	**fatty acid catabolic process**	HADHB; HADHA; MCEE; ACAA2; BDH2; AUH; PCCA; PCCB; HSD17B4; ACADS; ACAT1	1.92E-05
GO:0006635	*fatty acid beta-oxidation*	HADHB; HADHA; ACADVL; ACAA2; BDH2; AUH; ETFDH; HSD17B4; ACADS; ACAT1	1.92E-05
GO:0019395	*fatty acid oxidation*	HADHB; HADHA; ACAA2; BDH2; AUH; HSD17B4; ACADS; ACAT1	8.63E-04
GO:0016101	*diterpenoid metabolic process*	ALDH3A2; LRP1; ADH1B; AKR1C1; AKR1C3	0.0076
GO:0006692	*prostanoid metabolic process*	AKR1C1; AKR1C3; AKR1C2; CES1	0.0082
GO:0006693	*prostaglandin metabolic process*	AKR1C1; AKR1C3; AKR1C2; PTGS1; CES1	0.0095
GO:0009083	*branched-chain amino acid catabolic process*	ALDH6A1; HIBADH; MCCC1; ACAT1	0.0095
**GO:0035987**	**endodermal cell differentiation**	MMP14; COL4A2; MMP2; COL12A1; COL6A1; FN1; HMGA2; LAMB1	5.21E-05
GO:0001706	*endoderm formation*	MMP14; COL4A2; MMP2; COL12A1; COL6A1; FN1; HMGA2; LAMB1	8.06E-05
GO:0048048	*embryonic eye morphogenesis*	FBN2; EFEMP1; MFAP2; FBN1	0.0013
**GO:0045765**	**regulation of angiogenesis**	GRN; SP100; PLXND1; HSPB6; SERPINF1; HMGA2; PRKCA; THBS2; HSPG2; FGF2; DCN; MECP2; SFRP1; GPNMB; COL4A2; CYP1B1; EMILIN2; EMILIN1	5.81E-05
GO:1901342	*regulation of vasculature development*	SP100; SFRP1; PLXND1; GPNMB; FGF2	0.0095
**GO:0006027**	**glycosaminoglycan catabolic process**	IDUA; GLB1; GUSB; FGF2; GNS; SGSH	6.91E-05
GO:0030167	*proteoglycan catabolic process*	NAGLU; GLB1; IDUA; SGSH	3.71E-04
GO:0006026	*aminoglycan catabolic process*	IDUA; GUSB; GNS; CTBS; SGSH	6.65E-04
GO:0030203	*glycosaminoglycan metabolic process*	IDUA; HEXB; HEXA; GUSB; GNS; SGSH	0.0013
GO:0030163	*protein catabolic process*	CTSL; CTSK; LAMP2; TPP1; APOE; TCIRG1; CTSC; CTSB; RAB7A	0.0076
**GO:0030336**	**negative regulation of cell migration**	IGFBP5; LRP1; STC1; PTPRK; FGF2; SFRP1; COL3A1; CDH11; DAG1; CYP1B1; EMILIN2; EMILIN1; TIMP1; RECK; ENG	2.08E-04
GO:0030334	*regulation of cell migration*	GRN; TNXB; SERPINE2; PLXND1; TNC; STC1; PTPRK; FGF2; SDCBP; GPNMB; LMNA; DAG1; CYP1B1; EMILIN2; EMILIN1; ARSB; PDGFRB; PDGFRA; IGFBP5; HGF; PRKCA; LAMB1; MMP14; SFRP1; CDH11; RECK; ENG	6.04E-05
GO:2000146	*negative regulation of cell motility*	SFRP1; IGFBP5; CDH11; DAG1; CYP1B1; STC1; EMILIN2; FBLN1; EMILIN1; PTPRK; RECK; ENG	0.0013
GO:0010596	*negative regulation of endothelial cell migration*	MECP2; SP100; STC1; APOE; FGF2; DCN	0.0040
GO:0043535	*regulation of blood vessel endothelial cell migration*	MECP2; STAT5A; P2RX4; PRKCA; PRCP; APOE; FGF2	0.0044
**GO:0007040**	**lysosome organization**	SRPX; GRN; GAA; TMEM106B; ACP2; TPP1; LAMTOR1; ARSB	3.53E-04
GO:0080171	*lytic vacuole organization*	GRN; GAA; TMEM106B; ACP2; TPP1; LAMTOR1; ARSB	7.11E-04
**GO:0007034**	**vacuolar transport**	GRN; LRP1; NPC1; VPS13C; PSAP; TMEM106B; ARSB; VTI1B	4.74E-04

**GO:0042127**	**regulation of cell population proliferation**	KANK2; SLC35F6; COL18A1; TNXB; CD81; HP1BP3; TCIRG1; PTPRK; FGF2; CLU; MECP2; DPP4; SDCBP; GPNMB; FTH1; LMNA; CYP1B1; EMILIN2; IGFBP7; EMILIN1; TIMP1; TP53I11; TNS2; STAT5A; PDGFRB; PDGFRA; FN1; AKR1C3; AKR1C2; GREM1; BST1; SFRP1; DDAH1; CRLF1; ENG	6.28E-04
**GO:1903053**	**regulation of extracellular matrix organization**	EFEMP2; LRP1; LAMB1; LAMC1; NID1; DDR2	6.28E-04
**GO:0030301**	**cholesterol transport**	NPC1; OSBPL5; AKR1C1; APOC3; APOE; ABCA8; CLU; CES1	7.82E-04
GO:0043691	*reverse cholesterol transport*	APOC3; APOE; CLU; CES1	0.0082
**GO:0006123**	**mitochondrial electron transport, cytochrome c to oxygen**	NDUFA4; COX7A2; COX5A; COX7C; COX6B1	0.0011
GO:0045333	*cellular respiration*	NDUFB6; NDUFS6; NDUFA4; ETFDH; COX7A2; COX7C; COX5A; COX6B1	0.0095
**GO:0043062**	**extracellular structure organization**	MMP14; COL3A1; COL4A2; COL4A1; COL14A1; ADAMTSL4; MMP2; PXDN; TGFBI; RECK; THSD4	0.0011
**GO:0045229**	**external encapsulating structure organization**	MMP14; COL3A1; COL4A2; COL4A1; COL14A1; ADAMTSL4; MMP2; PXDN; TGFBI; RECK; THSD4	0.0012
**GO:0035581**	**sequestering of extracellular ligand from receptor**	FBN2; GREM1; LTBP1; FBN1	0.0013
**GO:0071379**	**cellular response to prostaglandin stimulus**	SFRP1; GNG2; AKR1C3; AKR1C2	0.0013
**GO:0097435**	**supramolecular fiber organization**	TMOD1; COL18A1; TNXB; COL14A1; LUM; COL12A1; LTBP2; LOXL1; THSD4; CST3; GREM1; MFAP4; COL3A1; EFEMP2; PXDN; CYP1B1; EMILIN1; DDR2; SNCA	0.0015
**GO:0010466**	**negative regulation of peptidase activity**	CST3; SERPINE2; LRP1; SERPINF1; TIMP2; SERPING1; TIMP1; CTSB	0.0016
GO:0010951	*negative regulation of endopeptidase activity*	CST3; SERPINE2; SERPINF1; TIMP2; SERPING1; TIMP1; RECK	0.0099
**GO:0038202**	**TORC1 signaling**	LAMTOR2; LAMTOR1; LAMTOR3; LAMTOR5	0.0019
**GO:2001044**	**regulation of integrin-mediated signaling pathway**	CD63; LAMB1; LAMC1; TIMP1; NID1	0.0019
**GO:0010874**	**regulation of cholesterol efflux**	LRP1; APOE; ABCA8; LAMTOR1; PLTP; CES1	0.0021
GO:0010875	*positive regulation of cholesterol efflux*	LRP1; ABCA8; APOE; PLTP; CES1	0.0033
GO:0032376	*positive regulation of cholesterol transport*	LRP1; APOE; ABCA8; PLTP; CES1	0.0099
**GO:0044273**	**sulfur compound catabolic process**	NAGLU; IDUA; GLB1; GNS; SGSH	0.0022
**GO:0008284**	**positive regulation of cell population proliferation**	PDGFRB; SLC35F6; PDGFRA; GRN; TNXB; CD81; FN1; AKR1C3; AKR1C2; HMGA2; LAMB1; TCIRG1; LAMC1; FGF2; MECP2; DPP4; GREM1; BST1; SDCBP; SFRP1; TMEM119; TIMP1; CRLF1; DDR2	0.0024
**GO:0008203**	**cholesterol metabolic process**	CYP27A1; ACAA2; OSBPL5; GLB1; AKR1D1; APOE; OSBPL1A; CES1	0.0030
GO:0016125	*sterol metabolic process*	CYP27A1; OSBPL5; GLB1; CYP1B1; APOE; OSBPL1A; CES1	0.0071
GO:0008206	*bile acid metabolic process*	CYP27A1; NPC1; AKR1C1; AKR1D1; OSBPL1A	0.0099
GO:1902652	*secondary alcohol metabolic process*	CYP27A1; OSBPL5; GLB1; APOE; OSBPL1A; CES1	0.0099
**GO:0060429**	**epithelium development**	KANK2; CPT1A; BDH2; ANXA4; AKR1C1; TNC; DAG1; AKR1C2; VDAC1; TPP1; CES1; CTSB	0.0033
**GO:0006508**	**proteolysis**	CTSA; CFH; CPQ; HGF; MMP2; HTRA1; PCOLCE; ECE1; AEBP1; DPP4; MMP14; CTSL; ANPEP; CTSK; CPE; TPP1; CTSC; CTSB	0.0056
**GO:0010715**	**regulation of extracellular matrix disassembly**	CST3; DPP4; LRP1; DDR2	0.0060
**GO:1900221**	**regulation of amyloid-beta clearance**	LRPAP1; LRP1; APOE; CLU	0.0071
**GO:0060393**	**regulation of pathway-restricted SMAD protein phosphorylation**	GREM1; SDCBP; TWSG1; TNXB; LRP1; EMILIN1; ENG	0.0076
**GO:0007097**	**nuclear migration**	LMNA; SYNE3; LMNB2; LMNB1	0.0082
**GO:0007041**	**lysosomal transport**	SCARB2; GRN; LAMP1; NPC1; LRP1; PSAP; TMEM106B; ARSB; RAB7A	0.0087

**GO:0035909**	**aorta morphogenesis**	PDGFRB; EFEMP2; LRP1; ENG	0.0095
**GO:0007160**	**cell-matrix adhesion**	TIMM10B; CD63; COL3A1; ITGA2; CDH11; FN1; EMILIN1; PTPRK; NID2	0.0099

Table S3. Gene ontology biological process terms overrepresented in the DA protein set with reduced abundance in 3i-VFE compared to VFE, related to Figure 4. Terms were identified using the Enrichr algorithm based on a Benjamini Hochberg-adjusted *p*-value < 0.01 and minimum of 4 protein identifications per pathway, then postprocessed for redundancy using the REVIGO semantic similarity algorithm. Data are presented in order of ascending adjusted *p*-value of the representative term (in bold) for each cluster; nested terms are in gray and italicized.

Term	Description	Proteins	Adjusted *p*-value
**GO:0030239**	**myofibril assembly**	PDGFRB; ACTN2; TPM1; KLHL41; TTN; CSRP2; MYH3; TNNT1; MYL2; TNNT2; TNNT3; FLNC; MYL9; MYH7	1.42E-14
GO:0045214	*sarcomere organization*	CSRP2; MYH3; TNNT1; TNNT2; TPM1; TNNT3; FLNC; TTN; MYH7	1.81E-09
GO:0007517	*muscle organ development*	TRIM72; TAGLN; SGCD; MYH3; DES; LAMA2; ITGA7; VAMP5	9.02E-06
**GO:0006941**	**striated muscle contraction**	TNNC1; TPM1; KLHL41; TTN; MYL4; MYH3; TNNT1; MYL2; TNNT2; TNNT3; MYH8; TNNI1; MYH7	7.08E-12
GO:0006936	*muscle contraction*	TPM2; TPM1; KLHL41; TTN; MYH2; MYH3; DES; TNNT2; MYH8; MYH4; CRYAB; SNTB1; MYH7	1.81E-09
GO:0003009	*skeletal muscle contraction*	MYH3; TNNT1; TNNC1; TNNT3; MYH8; TNNI1; MYH7	4.57E-08
**GO:0097435**	**supramolecular fiber organization**	CRTAP; MARCKSL1; SH3KBP1; COL14A1; TPM2; TPM1; STMN2; KLHL41; LOXL2; CNN3; COL1A1; GREM1; TPPP3; COL3A1; COL1A2; DES; COL5A2; SERPINH1; XIRP1; CYP1B1; ARHGEF2; VIM; MYL9	4.51E-11
GO:0030199	*collagen fibril organization*	COL1A1; GREM1; CRTAP; COL3A1; COL1A2; COL14A1; COL5A2; SERPINH1; CYP1B1; LOXL2	1.81E-09
GO:0030198	*extracellular matrix organization*	CRTAP; COL14A1; LAMC1; LOXL2; COL1A1; GREM1; MMP14; COL3A1; COL1A2; COL5A2; SH3PXD2B; SERPINH1; CYP1B1; COL8A1	2.75E-07
**GO:0033275**	**actin-myosin filament sliding**	MYH2; MYH3; TNNT2; TPM1; MYH8; MYH4; MYH7	1.81E-09
GO:0030049	*muscle filament sliding*	MYH3; TNNT2; TPM1; MYH8; MYH4; MYH7	2.83E-08
**GO:0031032**	**actomyosin structure organization**	CSRP2; MYH3; ACTN2; TNNT1; TNNT2; TPM1; TNNT3; FLNC; MYL9; KLHL41; MYH7; TTN	2.38E-09
GO:0007015	*actin filament organization*	ACTA1; MARCKSL1; ACTC1; SH3KBP1; TPM2; DPYSL3; TPM1; XIRP1; ARHGEF2; CNN3; TTN	1.70E-05
**GO:0060047**	**heart contraction**	MYL4; SGCD; ACTC1; TNNC1; MYL2; TNNT2; TPM1; TTN; MYH7	2.91E-08
GO:0060048	*cardiac muscle contraction*	MYL4; TNNC1; MYL2; TNNT2; TPM1; TTN; MYH7	2.95E-06
**GO:0006937**	**regulation of muscle contraction**	TNNT1; TNNC1; MYL2; TPM1; TNNT3; TNNI1; MYBPH; MYL9	3.76E-08
GO:0006942	*regulation of striated muscle contraction*	MYL2; TNNT3; TNNI1; MYBPH	0.0045
GO:1903522	*regulation of blood circulation*	DES; CELF2; TNNT2; TPM1	0.0075
**GO:0110011**	**regulation of basement membrane organization**	LAMA2; LAMB2; LAMB1; LAMC1	4.43E-06
**GO:0035987**	**endodermal cell differentiation**	MMP14; COL6A1; FN1; COL8A1; ITGA7; LAMB1	3.95E-05
GO:0001706	*endoderm formation*	MMP14; COL6A1; FN1; COL8A1; ITGA7; LAMB1	8.01E-05
**GO:0055008**	**cardiac muscle tissue morphogenesis**	TNNC1; MYL2; TNNT2; TPM1; TTN; MYH7	3.95E-05
GO:0003229	*ventricular cardiac muscle tissue development*	TNNC1; MYL2; TNNT2; TPM1; MYH7	2.35E-04
GO:0055010	*ventricular cardiac muscle tissue morphogenesis*	TNNC1; MYL2; TNNT2; TPM1; MYH7	7.03E-04
GO:0003208	*cardiac ventricle morphogenesis*	TNNC1; MYL2; TNNT2; TPM1; MYH7	0.0018
**GO:0030155**	**regulation of cell adhesion**	LAMA2; PLAU; LAMB2; TPM1; SAA1; LPXN; PRKCA; LAMB1; LAMC1; TGM2	1.14E-04
**GO:0045785**	**positive regulation of cell adhesion**	LAMA2; LAMB2; TPM1; SAA1; PRKCA; LAMB1; LAMC1; TGM2	1.58E-04
**GO:1903053**	**regulation of extracellular matrix organization**	LAMA2; LRP1; LAMB2; LAMB1; LAMC1	1.58E-04
**GO:2001046**	**positive regulation of integrin-mediated signaling pathway**	LAMA2; LAMB2; LAMB1; LAMC1	2.40E-04
**GO:0010631**	**epithelial cell migration**	KANK2; DPP4; CYP1B1; LPXN; FSTL1; LOXL2	3.13E-04
GO:0043542	*endothelial cell migration*	DPP4; CYP1B1; LPXN; FSTL1; LOXL2	0.0015
**GO:0018149**	**peptide cross-linking**	COL3A1; FN1; F13A1; THBS1; TGM2	3.14E-04

**GO:0072359**	**circulatory system development**	PDLIM3; COL3A1; MYL2; SH3PXD2B; FN1; HSPG2; PDLIM4; PDLIM7; FBN1	3.35E-04
**GO:0007044**	**cell-substrate junction assembly**	ACTN2; FN1; LAMC1; THY1; FERMT2	3.54E-04
**GO:0007507**	**heart development**	PDLIM3; COL3A1; SGCD; MYL2; SH3PXD2B; FN1; PDLIM4; PDLIM7; FBN1; MYH7	3.78E-04
**GO:0010955**	**negative regulation of protein processing**	PLAU; CTSZ; SERPINE1; THBS1	5.09E-04
GO:0010755	*regulation of plasminogen activation*	PLAU; CTSZ; SERPINE1; THBS1	6.53E-04
**GO:0051149**	**positive regulation of muscle cell differentiation**	LAMA2; LAMB2; LAMB1; LAMC1	5.09E-04
**GO:0030335**	**positive regulation of cell migration**	COL1A1; PDGFRB; MMP14; DAB2; PLAU; FN1; PRKCA; LAMB1; ARHGEF2; SOD2; THBS1; FERMT2	7.20E-04
GO:0030334	*regulation of cell migration*	PDGFRB; NGFR; SERPINE1; TPM1; NEXN; PRKCA; LAMB1; THY1; SOD2; THBS1; COL1A1; MMP14; DAB2; PLAU; DPYSL3; CYP1B1; FERMT2	1.11E-04
GO:2000147	*positive regulation of cell motility*	COL1A1; PDGFRB; MMP14; DAB2; PLAU; PRKCA; LAMB1; SOD2; THBS1; FERMT2	0.0023
GO:0030336	*negative regulation of cell migration*	NGFR; COL3A1; LRP1; SERPINE1; DPYSL3; TPM1; CYP1B1; THY1	0.0055
**GO:0007229**	**integrin-mediated signaling pathway**	COL3A1; DAB2; ITGA1; FN1; ITGA7; THY1; FERMT2	8.15E-04
**GO:0034446**	**substrate adhesion-dependent cell spreading**	FN1; LPXN; LAMB1; LAMC1; FERMT2	9.58E-04
GO:0007160	*cell-matrix adhesion*	COL3A1; ACTN2; ITGA1; FN1; ITGA7; THY1; FERMT2	0.0030
**GO:0045597**	**positive regulation of cell differentiation**	COL1A1; DAB2; LAMA2; LAMB2; TGFB1I1; LAMB1; ARHGEF2; LAMC1; IL6ST; RBM24; FERMT2; LOXL2	9.58E-04
**GO:0014910**	**regulation of smooth muscle cell migration**	PDGFRB; LRP1; PLAU; SERPINE1	0.0011
**GO:2001044**	**regulation of integrin-mediated signaling pathway**	LAMA2; LAMB2; LAMB1; LAMC1	0.0011
**GO:0043588**	**skin development**	COL1A1; COL3A1; COL1A2; WNT5A; COL5A2; SPRR2B	0.0018
**GO:0031114**	**regulation of microtubule depolymerization**	MAP1B; MAP1A; STMN2; ARHGEF2	0.0025
**GO:0051147**	**regulation of muscle cell differentiation**	LAMA2; LAMB2; LAMB1; LAMC1	0.0025
**GO:0010718**	**positive regulation of epithelial to mesenchymal transition**	COL1A1; DAB2; TGFB1I1; FERMT2; LOXL2	0.0029
**GO:0043062**	**extracellular structure organization**	COL1A1; MMP14; COL3A1; COL1A2; COL14A1; COL5A2; COL8A1	0.0030
**GO:0045229**	**external encapsulating structure organization**	COL1A1; MMP14; COL3A1; COL1A2; COL14A1; COL5A2; COL8A1	0.0031
**GO:0001501**	**skeletal system development**	COL1A1; MMP14; COL1A2; WNT5A; SH3PXD2B; ALPL; TGM2; FBN1	0.0032
**GO:0007010**	**cytoskeleton organization**	DES; BIN1; SH3KBP1; PALLD; MICAL1; TPM1; THY1	0.0032
**GO:0010717**	**regulation of epithelial to mesenchymal transition**	GREM1; COL1A1; DAB2; TGFB1I1; FERMT2; LOXL2	0.0044
**GO:1901653**	**cellular response to peptide**	NGFR; LRP1; ARHGEF2; VIM; ICAM1	0.0055
**GO:0048522**	**positive regulation of cellular process**	PDGFRB; LAMA2; LAMB2; TPM1; FN1; PRKCA; LAMB1; LAMC1; THBS1; DPP4; GREM1; MMP14; MAP1A; SAA1; IL6ST; TGM2	0.0068
**GO:0033628**	**regulation of cell adhesion mediated by integrin**	DPP4; PLAU; SERPINE1; CYP1B1	0.0069
**GO:0046034**	**ATP metabolic process**	MYH3; MYH8; MYH4; MYH7	0.0075
GO:0009205	*purine ribonucleoside triphosphate metabolic process*	MYH3; MYH8; MYH4; MYH7	0.0064
**GO:0010038**	**response to metal ion**	TNNT2; MT1X; THBS1; LOXL2; TTN	0.0091

Table S4. Gene ontology biological process terms overrepresented in the DA protein set with reduced abundance in sphere compared to VFE, related to Figure 4. Terms were identified using the Enrichr algorithm based on a Benjamini Hochberg-adjusted *p*-value < 0.01 and minimum of 4 protein identifications per pathway, then postprocessed for redundancy using the REVIGO semantic similarity algorithm. Data are presented in order of ascending adjusted *p*-value of the representative term (in bold) for each cluster; nested terms are in gray and italicized.

Term	Description	Proteins	Adjusted *p*-value
**GO:0007015**	**actin filament organization**	FLII; CAPG; CNN3; CORO1C; TTN; CNN2; RAC2; XIRP1; PLS3; FLNA; VILL; PLS1; MARCKSL1; TPM3; ACTN1; TPM2; TPM1; DSTN; CD2AP; ACTA1; MYO1E; MYO1B; ACTC1; FAT1; ARHGEF2; DBN1; BCAR1	8.63E-13
GO:0031032	*actomyosin structure organization*	ACTN2; TMOD3; FLII; TPM1; KLHL41; TTN; PDLIM1; TJP1; CSRP2; MYH3; CSRP1; SYNPO2L; TNNT1; TNNT2; TNNT3; ZYX; MYH9; FLNC; MYH7	3.75E-11
**GO:0030239**	**myofibril assembly**	ACTN2; FLII; TMOD3; TPM1; KLHL41; TTN; CSRP2; MYH3; CSRP1; SYNPO2L; TNNT1; MYL2; TNNT2; TNNT3; FLNC; MYH7	1.43E-11
GO:0045214	*sarcomere organization*	CSRP2; MYH3; CSRP1; SYNPO2L; TNNT1; TNNT2; TPM1; TNNT3; FLNC; TTN; MYH7	2.39E-08
**GO:0006941**	**striated muscle contraction**	TNNC1; TPM1; ATP1B1; KLHL41; TTN; MYL4; MYH3; JSRP1; TNNT1; MYL2; TNNT2; TNNT3; MYH8; TNNI1; DMD; MYH7	3.23E-10
GO:0003009	*skeletal muscle contraction*	JSRP1; MYH3; TNNT1; TNNC1; TNNT3; MYH8; TNNI1; MYH7	1.59E-06
GO:0060047	*heart contraction*	MYL4; ACTC1; TNNC1; MYL2; TNNT2; TPM1; DMD; ATP1B1; TTN; MYH7	7.71E-06
GO:0060048	*cardiac muscle contraction*	MYL4; TNNC1; MYL2; TNNT2; TPM1; DMD; ATP1B1; TTN; MYH7	1.19E-05
GO:0006936	*muscle contraction*	TPM3; TMOD3; TPM2; TPM1; KLHL41; TTN; MYH2; MYH3; DES; TNNT2; MYH8; MYH4; CRYAB; MYH7	1.30E-05
**GO:0043588**	**skin development**	DSP; SPRR3; CLIC4; ANXA1; ITGB4; ITGA3; DHCR24; EVPL; TGM1; SCEL; CASP3; ITGA6; SPRR2B; SPRR1A; IVL; SPRR1B	5.07E-09
**GO:0097435**	**supramolecular fiber organization**	FLII; STMN2; SLAIN2; LOXL2; CNN3; CORO1C; CNN2; STMN1; RAC2; XIRP1; BID; KRT6B; DSP; MARCKSL1; TPM3; ACTN1; TMOD3; TPM2; TPM1; TBCB; KLHL41; CD2AP; MYO1E; CLIP1; MYO1B; DES; FAT1; EPPK1; ARHGEF2; DBN1; BCAR1	4.47E-08
**GO:0006695**	**cholesterol biosynthetic process**	ACLY; NSDHL; MVK; HMGCS1; CYP51A1; MSMO1; DHCR24; MVD; DHCR7; FDFT1	6.48E-08
GO:1902653	*secondary alcohol biosynthetic process*	ACLY; NSDHL; MVK; HMGCS1; CYP51A1; DHCR24; MSMO1; MVD; DHCR7; FDFT1	4.47E-08
GO:0016126	*sterol biosynthetic process*	ACLY; NSDHL; MVK; HMGCS1; CYP51A1; MSMO1; DHCR24; MVD; DHCR7; FDFT1	1.82E-07
GO:0008203	*cholesterol metabolic process*	SULT2B1; ACLY; NSDHL; MVK; HMGCS1; CYP51A1; MSMO1; MVD; DHCR24; DHCR7; FDFT1	9.26E-05
**GO:0009913**	**epidermal cell differentiation**	DSP; SPRR3; CLIC4; ANXA1; EVPL; TGM1; SCEL; CASP3; CTNNB1; SPRR2B; SPRR1A; IVL; SPRR1B	1.50E-07
GO:0030216	*keratinocyte differentiation*	TGM1; DSP; SPRR3; SCEL; CLIC4; ANXA1; CASP3; EVPL; SPRR2B; SPRR1A; IVL; SPRR1B	8.60E-08
**GO:0008544**	**epidermis development**	DSP; COL17A1; SPRR3; LAMB3; LAMA3; LAMC2; EVPL; KLK7; SCEL; FABP5; COL7A1; SPRR2B; SPRR1A; SPRR1B; SPRR2D	6.38E-07
**GO:0033275**	**actin-myosin filament sliding**	MYH2; MYH3; TNNT2; TPM1; MYH8; MYH4; MYH7	8.14E-07
GO:0030049	*muscle filament sliding*	MYH3; TNNT2; TPM1; MYH8; MYH4; MYH7	7.25E-06
**GO:0018149**	**peptide cross-linking**	TGM1; DSP; ANXA1; F13A1; EVPL; THBS1; SPRR1A; IVL; SPRR1B	1.96E-06
**GO:0003229**	**ventricular cardiac muscle tissue development**	TNNC1; MYL2; TNNT2; TPM1; PKP2; DSG2; HOPX; MYH7	1.42E-05
GO:0055008	*cardiac muscle tissue morphogenesis*	TNNC1; MYL2; TNNT2; TPM1; PKP2; TTN; MYH7	7.77E-04
GO:0055010	*ventricular cardiac muscle tissue morphogenesis*	DSP; TNNC1; MYL2; TNNT2; TPM1; PKP2; MYH7	0.0011

**GO:0043542**	**endothelial cell migration**	S100A2; GIPC1; PXN; LPXN; S100A12; MYH9; S100P; S100A9; LOXL2	6.20E-05
GO:0010631	*epithelial cell migration*	S100A2; GIPC1; PXN; LPXN; S100A12; S100P; S100A9; LOXL2	0.0012
**GO:0042060**	**wound healing**	DSP; TRIM72; MACF1; SPRR3; DST; CHMP1A; TPM1; CHMP2B; MYH9; EPPK1; EVPL; PPL	8.49E-05
**GO:0051017**	**actin filament bundle assembly**	LIMA1; MYO1B; CALD1; MICAL1; PLS3; PAWR; EZR; PLS1	1.23E-04
GO:0061572	*actin filament bundle organization*	LIMA1; MYO1B; CALD1; MICAL1; PLS3; PAWR; EZR; PLS1	1.23E-04
**GO:0007229**	**integrin-mediated signaling pathway**	CEACAM1; DST; ITGB4; ITGA3; ITGA1; ZYX; ITGA7; MYH9; ITGA6; ISG15; ITGB6; BCAR1	1.41E-04
**GO:0006937**	**regulation of muscle contraction**	TNNT1; TNNC1; MYL2; TPM1; TNNT3; TNNI1; MYBPH	4.98E-04
GO:0098911	*regulation of ventricular cardiac muscle cell action potential*	DSP; JUP; PKP2; DSG2	0.0067
**GO:0034109**	**homotypic cell-cell adhesion**	CSRP1; ACTN1; CEACAM5; HSPB1; FLNA; MYH9; MYL12A; TJP2	0.0012
**GO:0046034**	**ATP metabolic process**	MYH3; BAD; OLA1; MYH8; ATP1B1; MYH4; MYH7	0.00123
GO:0009205	*purine ribonucleoside triphosphate metabolic process*	MYH3; BAD; OLA1; MYH8; ATP1B1; MYH4; MYH7	9.44E-04
**GO:0086069**	**bundle of His cell to Purkinje myocyte communication**	DSP; JUP; PKP2; DSG2	0.0017
**GO:0006084**	**acetyl-CoA metabolic process**	ACLY; ACSS2; MVK; HMGCS1; MVD	0.0023
**GO:0045216**	**cell-cell junction organization**	DSP; TJP1; PRKCI; CTNND1; PKP2; DSG2; CSK; PKP3; TJP2	0.0033
**GO:0042981**	**regulation of apoptotic process**	RTKN; GSTP1; TRADD; FHL2; HSPB1; THBS1; BAG3; TRIM2; CASP3; FLNA; BID; PRKCI; ANXA1; ACTN2; BAD; ACTN1; PAWR; ASNS; SCRIB; DHCR24; ACTN4; OXSR1; YWHAZ; NME1; TJP1; GCLC; KRT18; BIN1; CEACAM5; RASA1; CTNNB1; ITGA6; CIAPIN1; CRYAB; BCAR1; MCM2	0.0037
**GO:0008610**	**lipid biosynthetic process**	ACLY; PCYT2; ACSS2; MVK; ACSL1; FASN; CYP51A1; MVD; FDFT1	0.0044
**GO:0007507**	**heart development**	OXSR1; CRIP1; PDLIM1; AKAP13; PDLIM3; PDLIM2; SYNPO2L; MYL2; SH3PXD2B; PKP2; PDLIM5; PDLIM4; PDLIM7; MYH7	0.0069
**GO:0022607**	**cellular component assembly**	YTHDF2; SLC2A1; PAWR; CAPG; YWHAZ; CD2AP; LIMA1; SLC9A3R1; MYO1B; CALD1; MICAL1; PKP2; PLS3; LPXN; DMD; EPPK1; EZR; PLS1	0.0069
**GO:0072359**	**circulatory system development**	PDLIM1; AKAP13; PDLIM3; PDLIM2; MYL2; SH3PXD2B; PKP2; OXSR1; PDLIM5; CRIP1; PDLIM4; PDLIM7	0.0075

Table S5. Human donor information, related to STAR Methods.

Donor	Age (years)	Sex	Cause of death	Postmortem interval (h)
Tissue processed for primary cell culture				
1	40	male	Complications following aortic valve repair	34
2	67	female	Blunt force trauma	10
3	83	female	End-stage renal disease	14
4	80	male	Polymicrobial peritonitis following intestinal perforation	22
5	18	female	Complications of sickle cell disease	15
6	46	male	Cervical spinal epidural abscess with lower extremity paralysis	16
7	76	male	Diffuse large B-cell lymphoma	15
8	71	male	Bronchopneumonia	20
Tissue processed for histology				
9	64	female	Acute lymphoblastic leukemia	64

Table S6. Primer sequences used for qRT-PCR, related to STAR Methods.

Gene symbol	Forward (5’-3’)	Reverse (5’-3’)
TP63	CTTGCCCAGGAAGAGACAGG	CATAAGTCTCACGGCCCCTC
PROM1	CAAGCCAGCCTCAGACAGAA	ATCTGTGGATGAAGGCTGCC
KIT	TCTGACGTCAATGCTGCCAT	TGGCAGTACAGAAGCAGAGC
CDH1	AGGCCAAGCAGCAGTACATT	GGATGTGATTTCCTGGCCCA
MUC1	AGCCACTTCTGCCAACTTGT	TGTCCGAGAAATTGGTGGGG
TUBB	GCCTTCCTCCACTGGTACAC	TCTGAGGGAGAGGAAAGGGG
SDHA	ACCTACTTCAGCTGCACGTC	CTCTCCACGACATCCTTCCG
